# Bioactive Potential of Algae and Algae-Derived Compounds: Focus on Anti-Inflammatory, Antimicrobial, and Antioxidant Effects

**DOI:** 10.3390/molecules29194695

**Published:** 2024-10-03

**Authors:** Maima Matin, Magdalena Koszarska, Atanas G. Atanasov, Karolina Król-Szmajda, Artur Jóźwik, Adrian Stelmasiak, Monika Hejna

**Affiliations:** 1 Department of Biotechnology and Nutrigenomics, Institute of Genetics and Animal Biotechnology of the Polish Academy of Sciences, 05-552 Jastrzębiec, Poland; m.matin@igbzpan.pl (M.M.); m.koszarska@igbzpan.pl (M.K.); atanas.atanasov@dhps.lbg.ac.at (A.G.A.); k.krol@igbzpan.pl (K.K.-S.); aa.jozwik@igbzpan.pl (A.J.); 2 Ludwig Boltzmann Institute Digital Health and Patient Safety, Medical University of Vienna, 1090 Vienna, Austria; 3 Department of Technique and Food Development, Institute of Human Nutrition Sciences, University of Life Sciences of Warsaw, 02-787 Warsaw, Poland; adrian_stelmasiak@sggw.edu.pl

**Keywords:** anti-inflammatory, antioxidant, antimicrobial, micro and macroalgae, bioactive compounds, therapeutic applications

## Abstract

Algae, both micro- and macroalgae, are recognized for their rich repository of bioactive compounds with potential therapeutic applications. These marine organisms produce a variety of secondary metabolites that exhibit significant anti-inflammatory, antioxidant, and antimicrobial properties, offering promising avenues for the development of new drugs and nutraceuticals. Algae-derived compounds, including polyphenols, carotenoids, lipids, and polysaccharides, have demonstrated efficacy in modulating key inflammatory pathways, reducing oxidative stress, and inhibiting microbial growth. At the molecular level, these compounds influence macrophage activity, suppress the production of pro-inflammatory cytokines, and regulate apoptotic processes. Studies have shown that algae extracts can inhibit inflammatory signaling pathways such as NF-κB and MAPK, reduce oxidative damage by activating Nrf2, and offer an alternative to traditional antibiotics by combatting bacterial infections. Furthermore, algae’s therapeutic potential extends to addressing diseases such as cardiovascular disorders, neurodegenerative conditions, and cancer, with ongoing research exploring their efficacy in preclinical animal models. The pig model, due to its physiological similarities to humans, is highlighted as particularly suitable for validating the bioactivities of algal compounds in vivo. This review underscores the need for further investigation into the specific mechanisms of action and clinical applications of algae-derived biomolecules.

## 1. Introduction

Algae represent eukaryotic unicellular or multicellular organisms occurring in fresh and saltwater reservoirs. Microalgae are microscopic unicellular organisms existing individually or in group formations, while macroalgae are multicellular organisms (seaweed), which are divided into green (*Chlorophyta*), brown (*Phaeophyta*), and red algae (*Rhodophyta*) [1]. Microalgae and macroalgae are highly abundant producers that could be exploited for their high therapeutic potential. Algae are widely studied as a source of anti-inflammatory, antimicrobial, and antioxidant compounds [2,3]. Such bioactive molecules, including polyphenols, carotenoids, lipids, and polysaccharides may exert beneficial effects on human and animal health [4,5].

Research suggests that algal extracts of both microalgae and macroalgae have the ability to reduce chronic inflammation by modulating signaling pathways, such as the mitogen-activated protein kinase (MAPK) nuclear factor kappa-B (NF-κB), (MAPK/NF-κB), phosphoinositide-3-kinase–protein kinase B (PI3K)/AKT, and nuclear factor-erythroid 2 related factor (Nrf2) pathways, and inhibit free radical production [6,7,8]. Such bioactivities underlay potential benefits in a range of diseases, and algae extracts are currently studied in different disease processes such as cardiovascular diseases, cancer, and neurodegenerative diseases. Bioactive compounds obtained from algae may thus have putative therapeutic or preventive potential in these diseases [9,10].

Algae extracts also show potential as an alternative to non-steroidal anti-inflammatory drugs (NSAIDs) containing substances such as ibuprofen, diclofenac, acetylsalicylic acid, etc., whose long-term use may lead to the development of side effects from the gastrointestinal tract, kidneys, and liver [11,12,13]. Bioactive compounds of algae, such as polyphenols, fucoitans, and polysaccharides, exhibit a number of anti-inflammatory properties [14,15]. Since inflammation is broadly involved in the pathogenesis of many diseases, algae have many potential applications in medicine, supporting the healing of wounds and burns, the treatment of heart disease, antiviral therapies, counteracting obesity, and the replacement of antibiotic therapy.

On this background, the aim of this comprehensive review was to provide an updated compilation of the bioactivities of algae and algae-derived biomolecules and to outline their anti-inflammatory, antimicrobial, antioxidant effects and therapeutic potential in the context of various health and disease states.

## 2. Bioactive Potential of Algae Extracts: Their Anti-Inflammatory Activity

**Figure 1:**
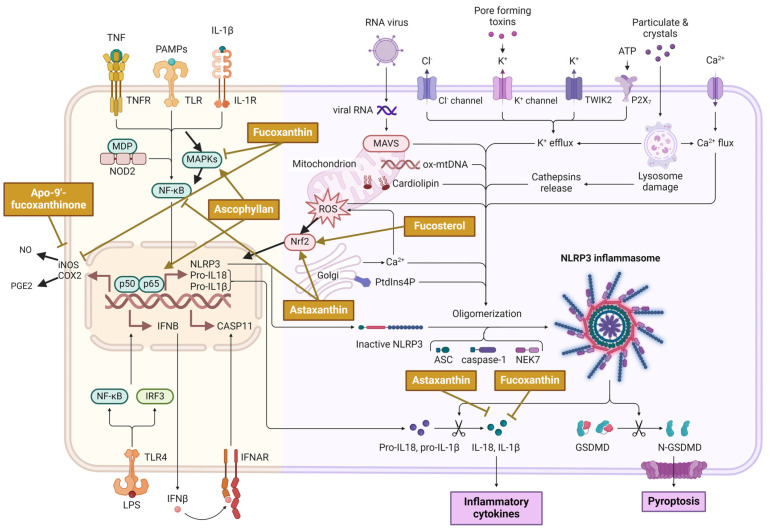
Key pro-inflammatory pathways and interference of selected algal compounds with these pathways (created with BioRender.com, on 15 May 2024).

Inflammation is the natural physiological reaction of the immune response to foreign stimuli that happens when the human body is exposed to different pathogens, toxic compounds, and damaged cells, ultimately triggering to persuade acute or chronic inflammatory reactions in various organs, causing tissue injuries or some immune diseases (Figure 1; [16,17]). As immune cells, macrophages are the most important mediators that produce pro-inflammatory enzymes such as inducible nitric oxide synthase (iNOS), cyclo-oxygenase-2 (COX-2), and various pro-inflammatory cytokines, such as tumor necrosis factor-α (TNF-α) and interleukins (IL-6, IL-1β). However, excessive induction and uncontrollable production of these mediators and cytokines can lead to higher inflammation and damage healthy cells, causing autoimmune disorders. Suppression of these important inflammatory mediators can serve as an effective therapeutic strategy to prevent inflammatory disorders [18].

Various algal compounds showed anti-inflammatory activity. For instance, Pangestuti et al. demonstrated that fucoxanthin attenuated oxidative stress and inflammation in amyloid-β42 (Aβ42)-induced BV2 microglial cells. Fucoxanthin showed no toxicity effect in the MTT cell viability test at any of the concentrations (5, 10, and 50 μM) on BV2 cell proliferation. Moreover, fucoxanthin was proven to attenuate pro-inflammatory cellular secretion by lowering the excretion of pro-inflammatory mediators, such as TNF-α, IL-6, IL-1β, and prostaglandin E2 (PGE2), along with the reduced expression of iNOS, COX-2, and by decreasing the phosphorylation of the MAPK pathway [19]. Another study disclosed the evaluation of the anti-inflammatory properties of apo-9′-fucoxanthinone (APO-9′) on the production of pro-inflammatory cytokines. To obtain cells for cell culture and cytokine production, bone marrow was collected from six-week-old female mice C57BL/6. An MTT assay was used to assess cell viability, and Western blot analysis was performed on RAW 264.7 cells previously treated with or without APO-9′ (20 µM) for 1 h prior to treatment with CpG DNA. The results showed that no changes were detected in the viability of the cell with the used concentration (up to 20 µM). The experimental results demonstrated that APO-9 exerted its activity in both dendritic cells and bone marrow-derived macrophages by inhibiting the secretion of pro-inflammatory cytokines in these sites. APO-9′ was also effective against PGE_2_ and nitric oxide (NO) synthesis in lipopolysaccharide-mediated inflammation against cell line RAW 264.7, where the NF-κB activation was regulated by suppressing IκB-α within macrophages [20].

On the other hand, pharmacological therapies used in the treatment of chronic inflammation include NSAIDs, glucocorticoids, anti-rheumatic drugs, biological response modifiers, and immunosuppressive drugs [21,22]. However, long-term use of these substances has a number of negative side effects such as nausea, heart attacks, strokes, kidney damage, allergic reactions, and headaches. The occurrence of a wide range of side effects, especially in the case of NSAIDs, has raised concerns about the use of these drugs [23].

Therefore, alternatives to existing pharmacological therapies are being pursued that will be equally effective in extinguishing inflammation while minimizing the risk of side effects [24]. In such drug discovery efforts, molecules derived from nature have been a continuous source for the development of novel therapeutics [25,26].

### 2.1. Influence of Algae Extracts on Anti-Inflammatory Response

Inflammatory response is a highly homeostatic defense mechanism of the immune system, which results in a cascade of events aimed at eliminating the cause of the immune response [27]. It activates various cells and molecules, including immune cells, cytokines, chemokines, and prostaglandins [5]. The inflammatory response is regulated by a complex network of signaling pathways involving both innate and adaptive immune mechanisms and can be acute or chronic depending on the duration and intensity of the stimulus [4,9]. Signal pathways, including the NF-κB, MAPK, Janus kinase/signal transducer and activator of transcription (JAK-STAT), and 3-phosphoinositide kinase, as well as the protein kinase AKT, participate in the regulation of the inflammatory response and play a significant role in a variety of physiological and pathological processes [8]. While acute inflammation is a normal and important part of the immune response, chronic inflammation can lead to tissue damage and induce many chronic diseases such as diabetes, rheumatoid arthritis, insulin resistance, cardiovascular diseases, neurodegenerative disorders, and cancer [28,29]. Algae contain bioactive compounds, including phycocyanins, polysaccharides, fatty acids (ratio n–3/n–6), terpenoids, and polyphenols, and show potential as a natural alternative to traditional drug therapies in the treatment of inflammatory conditions due to their ability to modulate inflammatory stress pathways, possibly with less significant side effects [9,10,30]. Along this line, recent studies highlight specifically the anti-inflammatory effects of novel algal-derived polysaccharides, such as those from *Saccharina japonica*, which have been shown to inhibit key inflammatory pathways including NF-κB and MAPK, offering potential as alternative treatments to NSAIDs without the associated gastrointestinal side effects [31].

#### 2.1.1. Regulation of Inflammatory Mediator Production in Macrophages

The role of macrophages in inflammation is undeniable due to their crucial role in initiating, developing, and resolving the inflammatory state. Macrophages regulate the course of inflammation by controlling the production of inflammatory mediators such as interleukins (ILs), TNF-α, reactive oxygen species (ROS), NO, and PGE_2_ [31,32]. Suppression of macrophages or the mediators produced by them is a key mechanism that enables tissue repair during inflammation. Numerous extracts from micro- and macroalgae were demonstrated to be able to modulate the production of inflammatory mediators while having low cytotoxicity for cells (Table 1). Under normal physiological conditions, the production of cytokines (pro-inflammatory, anti-inflammatory) is in a state of homeostasis; however, an increase in the level of pro-inflammatory cytokines in the body leads to the initiation of the inflammatory response [33].

In general, cytotoxicity of algal extracts has often been observed across different human cell lines, highlighting the varying effects based on the algal species and extraction methods. For example, *Cystoseira crinita* extracts induced significant cytotoxic effects against HCT15 and MCF7 cell lines, with the chloroformic extract in particular inducing a loss of cell viability with IC50 values of 41 and 37 μg/mL, respectively [34]. Similarly, *Cystoseira compressa* extracts (chloroform, ethyl acetate, and methanol) showed IC50 values ranging from 27 to 130 µg/mL against A549, HCT15, and MCF7 human cell lines [35]. In addition, extracts from *Amphiroa zonata* exhibited murine leukemic cells L1210 in the concentration range from 15 to 375 µg/mL, while cytotoxicity was not found in non-cancer human fibroblast HDF and murine non-cancer NIH-3T3 cells [36]. These studies suggest cell type-specific therapeutic windows for relevant algal extracts, offering the potential for both anticancer and anti-inflammatory applications while maintaining low toxicity in non-cancerous cells.

Extracts from microalgae containing bioactive compounds have been revealed to suppress the expression of pro-inflammatory cytokines TNF-α, IL-1β, and IL-6 while increasing the production of anti-inflammatory cytokine IL-10 in the lipopolysaccharide (LPS)-induced macrophages cell line (RAW 264.7) [4,29,37]. For example, Dai et al., in their study, showed the anti-inflammatory effect of *Coccomyxa gloeobotrydiformis* polysaccharide (CGD) on LPS-induced inflammation in the RAW 264.7 macrophage cell line. For this purpose, NO assay was used to determine NO production. The secretion of cytokines (TNF-α, IL-6, IL-1β, and IL-10) and inflammatory mediators (PGE2, iNOS, and COX-2) as well as signaling pathways related to inflammation (MAPK/NF-κB, PI3K/AKT/JNK, JAK/STAT and Nrf2/HO-1) was determined by Western blot. CGD polysaccharide did not show toxicity even at high concentrations. By reducing the expression of iNOS and COX-2, the expression of PGE2 and the production of NO, induced by LPS, were suspended. The secretion of pro-inflammatory cytokines such as TNF-α, IL-6, and IL-1β decreased, while the level of IL-10 increased. Studies have shown that CGD enhances the anti-inflammatory pathway Nrf2/HO-1 while, at the same time, weakens inflammatory signaling pathways such as JAK/STAT or MAPK/NF-κB [4]. 

Importantly, recent studies have shown that diverse algal extracts, such as *Hydropuntia cornea* and *Gracilariopsis longissima*, do not exhibit cytotoxicity in human keratinocytes and gingival fibroblasts at concentrations below 100 μg/mL. Additionally, these extracts were non-cytotoxic even at concentrations as low as 5 μg/mL in RAW 264.7 macrophages, where they promote cytokine production, including TNF-α and IL-6 [38]. Lipids, polysaccharides, and phenols contained in extracts from macroalgae also effectively suppress the expression of pro-inflammatory cytokines TNF-α, IL-1β, INF-γ while increasing the production of anti-inflammatory cytokines such as IL-10 and TGF-β in LPS-induced (RAW 264.7 and THP-1) macrophage cells [29,39,40,41]. Ku et al. examined two species of blue-green algae (BGA), such as Nostoc commune var. *Sphaeroides Kützing* (NO) and *Spirulina platensis* (SP). Experiments were performed on the RAW 264.7 cell line, mouse bone marrow-derived macrophages (BMM), and splenocytes from apolipoprotein E knockout mice (apoE −/−) fed BGA. Algal lipid extracts (NOE and SPE, respectively) reduced the secretion of pro-inflammatory cytokines (TNF-α, IL-1β, and IL-6) in macrophages and splenocytes by inhibiting the NF-κB pathway [29]. Another group investigated the protective effect of 3-bromo-4,5-dihydroxybenzaldehyde (BDB) isolated from *Polysiphonia morrowii* on TNF-α/interferon (IFN)-γ-stimulated inflammation and skin barrier deterioration in HaCaT keratinocytes. Similar to the previous study, the results also showed that BDB reduced the expression of inflammatory cytokines (IL-6, IL-8, IL-13, IFN-γ, TNF-α). In a dose-dependent manner, BDB reduced the intracellular ROS level, thereby increasing cell viability [33]. Lipid extracts from red algae *Porphyra dioica*, *Palmaria palmata*, *Chondrus crispus*, and microalga (*Pavlova lutheri*) also showed anti-inflammatory potential in LPS-stimulated human THP-1 macrophages. In the study, THP-1 macrophages were exposed to lipid extracts for 24 h. All extracts inhibited the expression of several inflammatory genes in THP-1 (TLR1, TLR2, TLR4, TLR8, TRAF5, TRAF6, TNFSF18, IL6R, IL23, CCR1, CCR4, CCL17, STAT3, MAP3K1) [34]. Another tested algae that demonstrated attenuation of the inflammatory process was *Turbinaria decurrens*. The compound extracted from this alga was sulphated polygalactofucan SPTd-2 which demonstrated inhibition of the production of pro-inflammatory cytokines such as TNF-α, TGF-β, Il-2, IL-1β, IFN-γ in human monocytic THP-1 cells [35]. 

NO and PGE_2_ are important inflammatory mediators whose expression is regulated by inducible iNOS and COX-2, respectively [42]. Bioactive compounds contained in algae are able to inhibit the mRNA expression of COX-2 and iNOS as well as the production of PGE_2_ and NO in both LPS-stimulated mouse RAW 264.7 macrophages and human THP-1 macrophages [39,41,43]. Recent investigations into *Spirulina platensis* have demonstrated that its bioactive compounds significantly inhibit the production of pro-inflammatory cytokines, such as TNF-α, while enhancing the expression of anti-inflammatory cytokines like IL-10, thus showcasing its potential as a therapeutic agent in inflammation-related diseases [9]. Interestingly, on some occasions, stimulation of the production of NO and pro-inflammatory cytokines (TNF-α and G-CSF) in macrophages has also been observed, for example, upon incubation with polysaccharides from *Ascophyllum nodosum.* The effects of ascophyllan, fucoidan (A-fucoidan) derived from *Ascophyllum nodosum*, and fucoidan from Sigma (S-fucoidan) as fucoidan derived from another source (*Fucus vesiculosus*) on RAW 264.7 mouse macrophage cell line cells were tested. While ascophyllan and A-fucoidan did not show cytotoxicity on RAW 264.7 cells at concentrations up to 1000 lg/mL, S-fucoidan showed concentration-dependent cytotoxic effects. In RAW 264.7 cells treated with ascophyllan, NO levels were significantly higher compared to those treated with fucoidan. The range of concentrations tested was from 0 to 200 lg-mL. TNF-α and granulocyte colony-stimulating factor (G-CSF) secretion from RAW 264.7 cells was higher under the influence of ascofilan compared to fucoidan [38].

**Table 1:** Effect of different bioactive ingredients of algal extracts on the production of pro- and anti-inflammatory mediators and their influence on macrophage viability.

Bioactive Constituents	Algae Species	Bioactive Agent	Cell Model	Effects of Algae Extracts	Cytotoxicity	Reference
Lipids/ pigments	*Nostoc commune var. sphaeroides*	Palmitic acid, palmitoleic acid, linoleic acid, linolenic acid, triacylglycerol, wax esters/steryl esters, and moreover pigments.	LPS-induced RAW 264.7 macrophages.	Inhibition of TNF-α, IL-6, and IL-1β mRNA expression. Inhibition of PGE2 and NO.	Non-cytotoxic in the tested range.	[23,29]
	*Palmaria palmata*	Phospholipids, galactolipids, and three polar lipids (sulfoquinovosyl diacylglycerol (SQDG)) and two phosphatidylglycerols.	LPS-induced RAW 264.7 macrophages.	Inhibition of NO production.	Non-cytotoxic in the tested range.	[44]
	*Aurantiochytrium mangrovei*	Squalene, moreover astaxanthin, and canthaxanthin (pigments).	LPS-induced RAW 264.7 macrophages.	Inhibition of IL-6, IL-1β, and TNF-α.	Cytotoxic at a concentration of 1/500 to 1/100.	[45]
	*Palmaria palmata*	Fatty acids (n-3 PUFA), and pigments (chlorophyll a, β-carotene, and fucoxanthin).	LPS-induced THP-1 macrophages.	Suppression of IL-6 and IL-8 production.		[40]
						
	*Haematoccus* *pluvialis*	Carotenoids (astaxanthin).	LPS-induced 7 mice bone marrow-derived macrophages.	Inhibition of TNF-α, IL-6 and IL-1β mRNA expression.	-	[46]
	*Phyllospora comosa* *Ulva* sp.	Carotenoids (fucoxanthin). Caretonoids (β-carotene, lutein).	LPS-induced RAW 264.7 macrophages.	Inhibition of NO production.	Low cytotoxicity in ETOAc extracts, high toxicity in DCM and BuOH.	[39,47]
Protein–pigment complex	*Chlorella* *pyrenoidosa*	Chlorophyll, β-carotene, lutein, zeaxanthin, alpha-linolenic acid, sporopollenin.	LPS-induced RAW 264.7 macrophages.	Suppression of TNF-α, IL-6, IL-10. Reduced the NO production.	Non-cytoxicity in the range of 0 to 500 μg /mL.	[37,48]
Phenols	*Polysiphonia* *morrowii* *Ecklonia stoloonifera* *Laminaria japonica* *Ulva lactuca*	3,4-dihydroxybenzaldehyde (DHB). Phlorofucofuroeckol A. Polyphenol-rich components (e.g., Ferulic acid, Rosmarinic acid, Gallic acid, Kaempferol, Epigallocatehin). Polyphenol-rich components (e.g., Rutin, Hesperidin, Quecertin, EpigallocatechinGallate).	LPS-induced RAW 264.7 macrophages.	Suppression of IL-1β, IL-6 and TNF-α. Inhibition of PGE2 and NO production. Inhibition of NO production, as well as the expression of IL-1, IL-6, TNF-α, iNOS, and COX-2. Elevated expression of the antioxidant enzymes HO-1 and NQO-1.	Non-cytotoxic in the tested range cytoprotective effect.	[6,31,32,39,49]
	*Ecklonia cava*	Phloroglucinol, dieckol.	LPS-induced the PMA-differentiated THP-1 macrophages.	Suppression of IL-1β, IL-6, and TNF-α. Inhibition of PGE2 and NO production.	Cytotoxic effect above 50 μM.	[32,43]
Polysaccharide	*Ascophyllum nodosum*	Ascophyllan.	RAW 264.7 macrophages (non-induced).	Stimulation of the production of NO, TNF-α, and G-CSF.	No significant cytotoxic effects up to 1000 μg/mL.	[50]
	*Phyllospora comosa* *Ulva* sp.	Fucoidans, alginates. Ulvan.	LPS-induced RAW 264.7 macrophages.	Inhibition of NO production.	Low cytotoxicity in ETOAc extracts, high toxicity in DCM and BuOH.	[39,47]
	*Coccomyxa* *gloeobotrydiforms*	Not mentioned.	LPS-induced RAW 264.7 macrophages.	Inhibition of TNF-α, IL-6, and IL-1β mRNA expression, increasing of IL-10 mRNA expression. Inhibition of PGE2 and NO production.	Non-cytotoxic in the tested range.	[4]
	*Turbinaria* *decurrens*	Sulfated polygalactofucan (SPTd-2).	LPS-induced THP-1 macrophages.	Suppression of IL-6, IL-2, INF-γ, IL-1β, TNF-α, IL-12, and increasing TGF-β. Inhibition of PGE2 and NO production.	-	[41]
	*Saccharina japonica*	Fucoidan.	LPS-induced RAW 264.7 macrophages	Inhibition of TNF-α, IL-L, and IL-1β secretion.	Non-cytoxicity in the range of up to 200 μg/mL.	[7]

#### 2.1.2. Modulation of MAPK/NF-κB Pathways in Macrophages by Algal Constituents

The MAPK and NF-κB pathways are key signaling pathways involved in the regulation of the inflammatory response in macrophages. Pro-inflammatory cytokines activate the MAPK pathway, leading to the phosphorylation and activation of transcription factors activator protein 1 (AP-1) and NF-κB. Activation of these pathways can further stimulate the production of inflammatory mediators, including cytokines IL-1β, IL-6, TNF-α, and iNOS [8,51].

NF-κB is the most important transcription factor involved in the activation of genes related to the regulation of the immune and inflammatory response. In mammals, the NF-κB factor includes five proteins, RelA (p65), RelB, c-Rel, p105 (p50), and p100 (p52), which form heterodimers and homodimers through the Rel homology domain [29,52]. NF-κB binds to IkBα and, by being located in the cytoplasm, its translocation to the cell nucleus is prevented. As a result of various signals, the enzyme IkB kinase (IKK) is activated, which phosphorylates IkBα, leading to its ubiquitination and release of NF-κB, which is then translocated into the nucleus. NF-κB then binds specifically to DNA, initiating the production of inflammatory mediators. Thus, the activation of the NF-κB pathway by pro-inflammatory factors leads to the translocation of p50-p56 dimers into the nucleus, which in turn leads to the genetic expression of iNOS and COX-2 [23,51]. The MAPK pathway transmits signals to the cell nucleus in response to inflammatory stimuli. MAPKs, including extracellular signal-regulated kinase (ERK), c-Jun NH2-terminal kinase (JNK), and p38, play roles in cell growth, proliferation, and apoptosis. MAPK signaling is upstream of NF-κB, contributes to the inflammatory response, and regulates the expression of related genes [23,53].

Different examples of bioactive compounds found in micro- and macroalgae extracts can successfully modulate MAPK/NF-κB pathways in vitro in macrophages (Table 2). Signal pathways can be successfully blocked by inhibiting the phosphorylation of p50/p65 and JNK, ERK, and p38 kinase by applying appropriate extract concentrations. Blocking the translocation of these molecules into the cell nucleus results in decreased transcription of inflammatory mediator genes. While most studies on algal ingredients have demonstrated inhibition of MAPK/NF-κB pathway activation in macrophages, there are also reports indicating stimulatory action. For example, in the previously mentioned article [38], a significantly greater activity of ascophyllan (isolated from the microalgae *Ascophyllum nodosum*) in inducing the secretion of TNF-α and G-CSF from RAW 264.7 cells was demonstrated compared to fucoidans in the concentration range from 3.1 to 50 lg/mL. A similar situation occurred in mouse peritoneal macrophages, where ascophyllan significantly induced NO and cytokine production more than fucoidans. Electrophoretic mobility shifts assay (EMSA) using NF-κβ and AP-1 consensus sequences confirmed the ability of ascophyllan to activate these transcription factors. Ascophyllan was also presented to increase the nuclear translocation of p65, the phosphorylation and degradation of IκB-α, and the levels of phosphorylated ERK, p38, and JNK in mouse macrophage RAW 264.7 cells [50]. Another research demonstrated that *Desmodesmus quadricauda* extracts exerted no cytotoxic effects on HepG2 cells at concentrations of 0.2 mg/mL, with observed DNA strand breaks at this non-cytotoxic concentration, highlighting the potential for safe therapeutic applications [54].

#### 2.1.3. Modulation of the PI3K/AKT and JAK/STAT Signaling Pathways in Macrophages by Algal Constituents

The JAK/STAT signaling pathway, which consists of JAKs, STATs, and diverse membrane receptors, regulates cellular processes such as survival, proliferation, and immune response [59]. JAK proteins (JAK1, JAK2, JAK3, TYK2) phosphorylate each other and the respective receptor. STAT binds to phosphorylated tyrosine residues and is phosphorylated by JAK. This activates the transcription of the target genes [60]. PIAS, PTPs, and SOCS represent negative regulators of this pathway [61]. In the context of inflammation, the JAK/STAT pathway plays an important role in signal transduction initiated by the activation of multiple cytokine receptors [62]. The JAK/STAT pathway interacts with the PI3K/AKT pathway, which is involved in the regulation of multiple processes, including cellular metabolism, cell growth, proliferation, and survival [63]. P13K/AKT also plays a role in the regulation of the inflammation response and the conversion of macrophages to the pro-inflammatory phenotype [64]. 

Several studies demonstrated modulation of the P13K/AKT and JAK/STAT signaling pathways in macrophages. Dai et al. (2018) analyzed the expression of JNK, PI3K, AKT, and STAT3 signaling molecules in RAW 264.7 mouse macrophages that were incubated with LPS in the presence of a polysaccharide extracted from the microalga *Coccomyxa gloeobotrydiformis* [4]. Studies have shown that the CGD polysaccharide was able to significantly inhibit MAPK signaling by reducing the phosphorylation of MAPKs (such as JNK, ERK1/2, and p38) in LPS-stimulated RAW 264.7 cells. The work showed decreased expression of PI3K protein, inhibition of AKT, JNK, and STAT3 phosphorylation compared to macrophages stimulated with LPS, which causes indirect or direct inhibition of signaling pathways such as MAPK/NF-κB, PI3K/AKT/JNK, and JAK/STAT and thus triggering anti-inflammatory effects [4]. In another study, Ye et al. (2020) evaluated fucoidan isolated from *Saccharina japonica* (SF6) against LPS-activated RAW 264.7 macrophages in terms of structure, anti-inflammatory activity, and potential molecular mechanisms. SF-6 significantly inhibited LPS-induced production of pro-inflammatory mediators and cytokines, including NO, iNOS, COX-2, TNF-α, IL-6, and IL-1β. It has also been proven that SP6 blocked previously LPS-induced inflammatory pathways, including NF-κB, MAPK, JAK-2, and the signal transducer and activator of transcription STAT1/3 pathways and thus have an anti-inflammatory effect [7,65]. SF-6 showed no cytotoxicity in the range up to 200 μg/mL [7]. Lee et al. (2009) investigated the effect of fatty acids isolated from *Gracilaria verrucosa* on JAK/STAT signaling by measuring STAT1 phosphorylation in LPS-stimulated RAW 264.7 macrophages [65]. Isolated fatty acids inhibited NO, TNF-α, and IL-6 production in a dose-dependent manner by inhibiting NF-κB activation and STAT1 phosphorylation in response to interferon-gamma, indicating their potential as anti-inflammatory agents [57].

#### 2.1.4. Modulation of the Nrf2 Pathway in Macrophages by Algal Constituents

NF-E2-related factor 2 (Nrf2) is a transcription factor that regulates oxidative stress and inflammatory responses. It has been disclosed to disrupt LPS-activated regulation in a mouse model of inflammation. Nrf2 inhibits inflammation by counteracting transcriptional upregulation of pro-inflammatory cytokine genes [4,66]. Under normal conditions, Nrf2 is preserved in the cytoplasm by binding to its regulator, a Kelch-like ECH-associated protein 1 (Keap1). Upon various stimuli, it translocates to the nucleus, where it binds to the promoter and initiates the transcription of cytoprotective protein-coding genes, such as NAD(P)H, GCLC, SRXN1, TXNRD1, HO-1, GST, UGT, Mrps, and KEAP1 [66,67].

As demonstrated by different research, HO-1 can inhibit the production of inflammatory mediators (NO, PGE_2_, IL-1β, IL-6, TNF-α) and exert a range of beneficial effects through antioxidant, anti-apoptotic, cytoprotective, immunosuppressive, and anti-inflammatory actions [68]. In this context, Dai et al. (2018) investigated the mechanism of action of polysaccharides isolated from *Coccomyxa gloeobotrydiformis* in relation to Nrf2/HO-1 signaling. The mechanism of CGD polysaccharide on Nrf2/HO-1 was investigated, and the study demonstrated that CGD significantly enhanced Nrf2 translocation to the nucleus from cytoplasm, which resulted in increased HO-1 expression in LPS-stimulated RAW 264.7 cells [4]. The phytosterol fucosterol, a lipid from *Padina boryana*, also exhibited a similar effect on activated PM (solid particles, particulate matter)-stimulated mouse macrophages RAW 264.7 [31]. In this study, the expression of inflammatory mediators and oxidative stress were upregulated in response to PM. PM induced increased NO production, which was accompanied by inflammatory mediators such as iNOS, COX-2, IL-6, IL-1β, TNF-α and prostaglandins such as PGE2. Both NF-κB and MAPK were involved in this process. 

However, treatment with fucosterol suppressed this effect, and Nrf2 and HO-1 expression were significantly activated in a dose-dependent manner compared to the control. Treatment with the phytosterol disrupted the Keap1/Nrf2 association, promoting its translocation to the nucleus and stabilizing Keap1 in the cytoplasm [31]. In another study, Sanjeewa et al. (2020) demonstrated that dieckol, a polyphenol from *Ecklonia cava* exhibited anti-inflammatory potential against PM-induced damage in mouse RAW 264.7 macrophages [32]. RAW 264.7 exposures to fine dust increased the levels of NO, PGE2, mRNA expression of inflammatory mediators (COX-2, iNOS) and the levels of pro-inflammatory cytokines, such as IL-6, IL-1b, TNF-α. The use of dieckol weakened the production of all of the above factors. The extract protected cells from damage by reducing intracellular ROS through increased production of superoxide dismutase (SOD) and the activation of the Nrf2/HO-1 pathway [32]. Similarly, another study revealed that the terpenoid astaxanthin produced on an industrial scale from the microalga *Haematococcus pluvialis* significantly inhibited LPS-induced boost of the expression of pro-inflammatory cytokines such as interleukin 6 (Il-6) and Il-1β in RAW 264.7 cells [46]. This effect was complemented by the suppression of NF-κB activation and an LPS-induced boost of ROS by astaxanthin and enhanced Nrf2 nuclear translocation. Additionally, it suppressed the expression of the ROS-generating enzyme NADPH oxidase 2 in RAW 264.7 macrophages. Moreover, experiments using bone marrow-derived macrophages from wild-type and Nrf2-deficient mice indicated that both Nrf2-dependent and Nrf2-independent mechanisms are involved in the anti-inflammatory and antioxidant effects of astaxanthin [46].

In conclusion, algae extracts and diverse algae-derived polysaccharides, phytosterols, and terpenoids have been revealed to induce Nrf2 activation, leading to an increase in the expression of the HO-1 protein in macrophages. This, in part, contributes to the observed anti-inflammatory and cytoprotective effects. 

## 3. Bioactive Potential of Algae Extracts: Regulation of Oxidative Stress in Macrophages by Algae Constituents

Macrophages are immune cells that play a crucial role in defending the body against invasion by pathogens and harmful substances. However, the production of ROS by macrophages is not just involved in the elimination of pathogens but can also cause damage to host tissues if not properly regulated [69]. To prevent such damage, macrophages have developed several mechanisms to regulate ROS production [46]. One way in which macrophages protect the body is by generating ROS aimed to eliminate pathogens through a process called the oxidative burst. However, excessive ROS production can lead to oxidative stress, which can damage cellular components and lead to inflammatory and other pathological states. To regulate oxidative stress, macrophages use diverse mechanisms to balance ROS production and neutralization [46,66]. One important mechanism is the activation of the nuclear factor erythroid 2-related factor 2 (Nrf2) pathway. Nrf2 is a transcription factor that binds to antioxidant response elements (AREs) in the promoter regions of target genes, leading to the induction of a wide range of antioxidant enzymes and other protective molecules. In response to oxidative stress, macrophages activate Nrf2 by preventing its degradation and promoting its translocation to the cell nucleus [70,71]. This can be achieved through various signaling pathways, including the MAPK pathway and the PI3K/Akt pathway. Once in the nucleus, Nrf2 activates the transcription of genes encoding antioxidant enzymes such as catalase, superoxide dismutase (SOD), and glutathione peroxidase (GPx), as well as other protective molecules such as heme oxygenase-1 (HO-1) [66,70]. 

Another mechanism through which macrophages regulate ROS production is through the activity of enzymes called NADPH oxidases (NOX). NOX enzymes are responsible for generating ROS in macrophages, and their activity is tightly regulated to prevent excessive ROS production [46]. In addition to the above-mentioned mechanisms, macrophages also utilize other mechanisms to regulate oxidative stress, such as the production of non-enzymatic antioxidants and the induction of autophagy, a process that removes damaged cellular components and reduces oxidative stress [46,55,72]. Overall, the regulation of oxidative stress in macrophages involves a complex interplay of signaling pathways and transcriptional regulation, which helps maintain a balance between ROS production and neutralization and prevents excessive oxidative damage [73].

Oxidative stress, associated with the overproduction of ROS in macrophages, is closely linked to the expression of pro-inflammatory genes. Fucoxanthin extracted from brown algae exhibits strong antioxidant effects. The study aimed to investigate the anti-inflammatory properties of fucoxanthin (FX) isolated from *Ishige okamurae* in RAW 264.7 cells previously stimulated with LPS. FX dose-dependently inhibited the production of iNOS, COX-2, NO, PGE2, and pro-inflammatory cytokines such as IL-1β, IL-6, and TNF-α. The downregulation of pro-inflammatory mediators in RAW 254.7 was mediated by inhibiting NF-κB activation and suppressing MAPK phosphorylation [44]. Recent work on the brown alga *Sargassum horneri* has revealed its potent antioxidant activity, which operates through the modulation of the Nrf2/HO-1 pathway, further enhancing cellular protection against oxidative stress by reducing the levels of ROS [7]. Another research study showed that LPS and t-butyl hydroperoxide (t-BHP) significantly increase ROS production in RAW 264.7, which is inhibited by the presence of the carotenoid. Induction of antioxidant genes such as Sod1, Gxp1, Gxp4, and Cat by this compound also contributes to the inhibition of cellular ROS accumulation [74]. The Nrf2/HO-1 pathway is an evolutionary conserved mechanism. In the cytosol, Nrf2 and Keap1 are bound to each other. Under stress conditions, Nrf2 rapidly dissociates and moves to the nucleus, where it binds to the ARE, resulting in the transcription of antioxidant genes [31,74]. Bioactive compounds such as fucoxanthin obtained from edible brown algae can target the Nrf2 pathway to protect against oxidative stress by increasing the expression of antioxidant genes. Therefore, it can be inferred that fucoxanthin-induced nuclear translocation of Nrf2 contributes to the reduction of ROS production and accumulation in RAW 264.7 macrophage cell models [74,75]. 

Additionally, *Ecklonia cava*, a brown alga, has been shown to possess significant antioxidant potential by reducing ROS accumulation in macrophages via upregulating Nrf2 expression, providing a promising approach for oxidative stress-related pathologies [6]. Farruggia et al. showed that astaxanthin exerts anti-inflammatory activity in two ways, by inhibiting the nuclear translocation of NFkB p65 and by reducing the accumulation of ROS in cells (dependently or not on NRF2). Another observation was that obese mice treated with astaxanthin had an altered sensitivity of splenic macrophages to LPS. This was also confirmed by in vitro studies [46]. In RAW 264.7 cells challenged by H_2_O_2_, increased viability and decreased intensity of ROS accumulation can be observed after incubation with astaxanthin, with the astaxanthin treatment restoring SOD activity and reducing malondialdehyde (MDA) levels [76]. 

Moreover, astaxanthin can be successfully used to counteract diseases caused by alcohol consumption associated with oxidative stress. This compound not only inhibits ROS production in ethanol-damaged macrophages in mice but also prevents a decrease in cellular NAD+ levels and increases SIRT1 activity [71]. Therefore, astaxanthin obtained from algae such as *Haematococcus pluvialis* or *Spirulina platensis* possesses certain antioxidant effects on oxidative damaged cells [46,76]. This study aimed to investigate the protective effect of astaxanthin on H_2_O_2_-induced damage in RAW 264.7 mouse macrophages by affecting the viability of these cells, ROS, and the mRNA expression of apoptosis factors. Astaxanthin at a concentration of 40M increased the viability of damaged cells, had a positive effect on the ROS level, restored SOD activity, and reduced MDA activity. These results indicated the antioxidant effect of astaxanthin in oxidatively damaged cells. Microscopic observation also revealed that astaxanthin inhibited the apoptosis of damaged cells, which resulted in cell protection. It was also found that as the concentration of astaxanthin increased, the expression of cell morphology and apoptosis factor mRNA were gradually restored [68]. 

Furthermore, another research study presented that fucosterol, a lipid from *Padina boryana* had antioxidant properties [31]. The expression of Nrf2 and Keap1 in the presence and absence of this phytosterol under solid particle stimulation (PM) conditions in mouse macrophages RAW 264.7 has been changed. Results of the analysis of the Nrf2/HO-1 pathway activation status suggested a decrease in ROS levels due to the action of the phytosterol on this pathway in macrophages. Nrf2 expression was upregulated in the presence of PM, and incubation with fucosterol had a significant stimulatory action compared to control, thus the key antioxidant factor, HO-1, was dose-dependently upregulated [31]. Overall, diverse bioactive compounds contained in algae such as phenols, lipids, and polysaccharides are capable of inhibiting iNOS mRNA expression and thus NO production in macrophages [39,41,43]. 

In summary, extracts from algae can modulate the gene expression related to macrophage oxidative stress by activating antioxidant response, inhibiting pro-inflammatory gene expression, and influencing relevant signal transduction pathways. These effects can lead to a reduction in oxidative stress and inflammatory state, which may have therapeutic benefits in various diseases associated with oxidative stress and inflammation, such as heart disease, diabetes, and cancer.

### Regulation of Apoptosis by Algal Constituents

**Figure 2:**
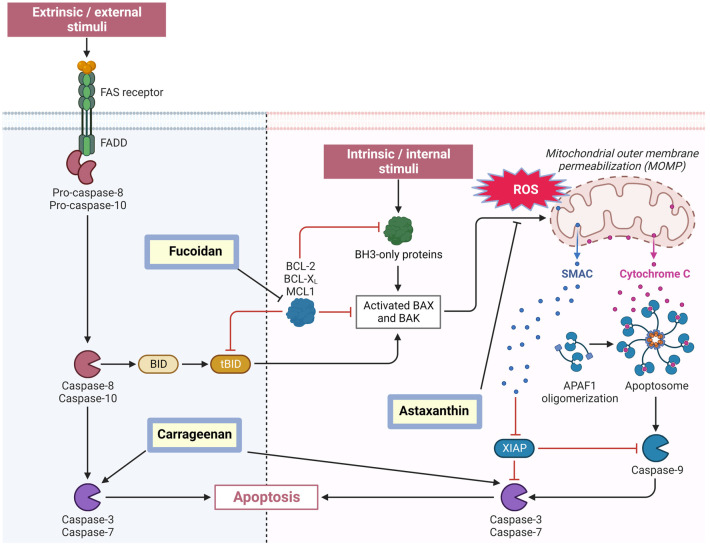
The basic external and internal signaling pathways lead to apoptosis and interference of selected algal compounds with these pathways (created with BioRender.com, on 10 June 2024).

Apoptosis, or programmed cell death, is an important process that ensures tissue homeostasis, developmental cell renewal and maintains a properly functioning life cycle [77]. In the context of cancer immunity, apoptosis plays a major role, with macrophages acting as key players in this process. Tumor-associated macrophages (TAMs) can either promote or inhibit tumor growth depending on their phenotype, while the clearance of apoptotic cancer cells by macrophages can shape anti-tumor immune responses [78]. Thus, macrophages are essential components of the tumor microenvironment and can significantly influence cancer progression. M1-like macrophages conventionally exhibit anti-tumor activity by promoting inflammation and T cell responses, while M2-like macrophages often support tumor growth through immunosuppression and angiogenesis [79]. In this context, algal constituents that can shift the balance towards M1 phenotypes or induce apoptosis in M2-like TAMs may enhance anti-tumor immunity. Regulation apoptosis in macrophages is a complex process involving multiple signaling pathways and molecular mechanisms [80]. Dysfunctions in apoptotic pathways, often induced by inflammation, cellular stress, and DNA degradation, can lead to serious autoimmune diseases, cancers, and cardiovascular-related disorders. In the context of cancer, the ability of tumor cells to evade apoptosis is a hallmark of the disease, and restoring apoptotic sensitivity is a key goal of many cancer therapies [72,81]. Both the external and internal signaling pathways can lead to cell death (Figure 2). The external pathway involves death receptors from the TNF superfamily, while the internal (mitochondrial) pathway is activated by stress factors such as oxidative stress or DNA damage [72,76]. 

Significant evidence suggests that diverse bioactive compounds from algae extracts are able to induce apoptosis, thereby regulating the proliferation and cell cycle of cancer cells [82,83]. For instance, a sulfated polysaccharide, such as fucoidan, isolated from brown algae such as *Fucus vesiculosus*, *Cladosiphon okamuranus*, and *Laminaria saccharina*, exhibited chemopreventive properties by scavenging free radicals. Fucoidan is able to regulate the mechanism of apoptosis in cancer cells by downregulating the Bcl-2 protein and upregulating Bax, increasing the permeability of the mitochondrial membrane and releasing cytochrome c [28,37,84]. It also induces apoptosis through the regulation of internal ROS levels [84]. Sulfated polysaccharides such as fucoidan regulate apoptosis in vitro in various cancer cell lines, including Human Leukemia (HL60), Burkitt’s lymphoma (HS-Sultan), and histiocytic lymphoma monocytes (U937), by regulating the caspase-3 and -7 pathway [85]. Importantly, some algal compounds, such as fucoidan, have demonstrated the ability to induce apoptosis in cancer cells while simultaneously activating macrophages. Thus, fucoidan from *Fucus vesiculosus* has been shown to induce apoptosis in cancer cells by downregulating Bcl-2 and upregulating Bax, while also enhancing the phagocytic activity and cytokine production of macrophages [86,87,88,89]. This dual action on both cancer cells and immune cells highlights the multi-target potential of algal constituents such as fucodain in cancer immunotherapy.

Another sulfated polysaccharide group is carrageenan obtained from red algae (*Rhodophyta*). κ-carrageenan exhibits anti-proliferate effects on breast cancer (MCF-7) and colon cancer (HT-29) cell lines inducing apoptosis through both the death receptor-dependent pathway with activation of caspase-3 and the mitochondrial pathway [90]. Furthermore, *kappa, iota, and lambda* carrageenans isolated from *Palisada perforata* were observed to significantly induce apoptosis in MCF-7 cells through increased caspase-3 activity and the upregulation of the bax: bcl-2 expression ratio [91,92]. Signs of induction of apoptosis were also found in a lung cancer cell line (A549) treated with polysaccharide isolated from *Acanthopora spicifera*, which caused cell membrane damage. A549 cells treated with the polysaccharide revealed apoptotic orange cells and red necrotic cells [93]. Moreover, the green microalga *Chlorococcum humicola*, due to its high level of bioactive compounds including fatty acids, carotenoids, sterols, and terpenes, exhibits anticancer and chemopreventive activity. The ability of *Chlorococcum humicola* extracts to alter the expression of Bcl-2 and Bax proteins in colon cancer cells (HT29) inducing the mitochondrial apoptotic pathway as well as the receptor-dependent pathway has been demonstrated [94]. Another example is the phytochemical obtained from *Gelidiella acrerosa*, which inhibited the proliferation of A549 cells and induced apoptosis by activating caspase-3 and altering the Bcl-2/Bax ratio [82]. Moreover, astaxanthin produced by co-fermentation of *Spirulina platensis* and *Saccharomyces cerevisiae* protects RAW 264.7 macrophages from oxidative damage induced by H_2_O_2_. Increased intensity of apoptosis and production of ROS, as well as activation of caspase-3 and caspase-9, were also effectively inhibited by treating damaged H_2_O_2_ macrophages with astaxanthin [76].

ROS regulates the process of apoptosis by directly decreasing the level of Bcl-2 and inducing Bax [95,96]. An extract from *Ulva fasciata* inhibited the growth of colon cancer cells (HCT116). The extract increased the ability of HCT116 mitochondria to produce hydrogen peroxide and superoxide anion, thereby increasing the level of ROS. This contributes to increased cytotoxicity on oxidative stress of cancer cells. The bioactive compounds contained in the *Ulva fasciata* extract changed the ratio between Bcl-2 and Bax proteins and activated caspases-3 and -9, thereby inducing HCT116 apoptosis through the mitochondrial pathway [95]. Another study presented that extracts from the brown algae *Colpomenia sinuosa* showed similar effects on HCT116 cells, inducing cell death by increasing ROS production. Extracts from *Colpomenia sinuosa* induced apoptosis through the mitochondrial pathway by changing the expression of Bcl-2 and caspase-3 [96]. 

The interaction between apoptotic cancer cells and macrophages is critical in shaping the immune response to tumors. Certain algal polysaccharides have been found to enhance the ability of macrophages to recognize and phagocytose apoptotic cancer cells. This increased clearance of apoptotic cells by macrophages can lead to enhanced antigen presentation and activation of anti-tumor T cell responses [97]. Along this line, the combination of apoptosis-inducing and immunomodulatory effects of diverse algal constituents makes them promising candidates for combination therapies with existing cancer immunotherapies such as immune checkpoint inhibitors.

In summary, significant evidence suggests that bioactive compounds from algae extracts are able to induce apoptosis, thereby controlling the proliferation and cell cycle of cancer cells. These compounds, such as fucoidan, carrageenan, and various other natural products found in different types of algae, exhibit the potential to modulate apoptotic pathways through mechanisms involving the regulation of Bcl-2 and Bax proteins, caspase activation, and regulation of ROS levels. By incorporating these elements, algal compounds offer a multifaceted approach to cancer therapy, potentially enhancing both direct cytotoxic effects on cancer cells and stimulating anti-tumor immune responses through modulation of macrophage function and apoptotic cell clearance.

## 4. Diversity of Bioactivities and Potential of Algae as Therapeutic Agents

Algae may have great potential as therapeutic agents due to the variety of biologically active compounds, such as polysaccharides, peptides, lipids, pigments, and polyphenols [23,29,39]. Algae extracts are rich in antioxidants that have a high potential to protect cells from damage caused by free radicals. Algae also contain compounds that have anti-inflammatory properties which help to reduce the inflammation process in the body, which can be beneficial in the treatment of arthritis, diabetes, cardiovascular diseases, bacterial infections, and chronic diseases such as cancer or Alzheimer’s disease [37,83,98,99]. Moreover, algae contain polysaccharides and peptides that promote wound healing and skin health [100].

### 4.1. Potential to Replace Antibiotics and Studies on Antimicrobial Activity to Counteract Infections 

Antibiotics are drugs that are designed to specifically kill or inhibit the growth of bacteria and are commonly used to treat bacterial infections. Nowadays, the overuse of antibiotic agents increases the emergence of multi-drug-resistant pathogenic bacteria, which has become a global problem [101,102,103,104]. Antibiotics are drugs that are also often used in animal husbandry to fight bacterial diseases. Unfortunately, their excessive and frequent application has resulted in the emergence of bacterial strains that are resistant to antibiotics, both in animals and in humans [105]. This phenomenon reduces the effectiveness of antibiotics in farm animals and increases the risk of transmitting resistant pathogens to humans [105,106]. Moreover, *Escherichia coli* is a common pathogen in pig farming and can cause a significant increase in morbidity and mortality in weaned piglets [106]. Farmers have difficulty dealing with this pathogen because antibiotics are ineffective against the toxins secreted by these bacteria. Thus, it is important to find effective alternatives to combat *Escherichia coli* [102] and reduce the overall use of antibiotics by searching for alternative solutions such as functional feed additives for farm animals [105,106].

One of the alternatives is algae, with a broad spectrum of bioactivities which hold potential in combating a wide range of pathogens (Table 3) [103]. Micro- and macroalgae extracts have revealed promising antibacterial properties in various studies targeting bacteria such as *Bacillus subtillis*, *Staphylococcus aureus, Escherichia coli*, and *Helicobacter pylori*. Botanical blends (100 mg/kg of BB1, 50 mg/kg, or 100 mg/kg of BB2) were dietary supplemented in sixty weanling pigs (7.17 ± 0.97 kg). Healthy weaned piglets were experimentally infected with a pathogenic *Escherichia coli* F18. Intestinal tissues and mucosa were collected for analyzing histology and gene expression. The results showed that botanical blends enhanced the performance and disease resistance of weaned pigs [107]. Frazzini et al. (2022) demonstrated that *Ascophyllum nodosum* showed the highest efficiency in inhibiting the growth of *Escherichia coli*. In this experiment, a commercially purchased dried meal of *Ascophyllum nodosum* was extracted using a 50:50 methanol and deionized water mixture. After centrifuging (15 min, 10,000 rpm, 4 °C) and filtering the supernatants (0.45 μm), a growth inhibition assay with *Escherichia coli* O138 was performed. Extracts were diluted in the medium at concentrations of 25%, 12.5%, 6%, 3%, and 1.5%. The growth rate of bacteria was estimated over six h by absorbance measurement [105]. Another study presented that the extracts from *Sphaerococcus coronopifolius* had effectiveness against methicillin-resistant *Staphylococcus aureus*. In this study, *Sphaerococcus coronopifolius* was collected from a water reservoir, then the algal material was freeze-dried. Further, extraction was then performed using vacuum liquid chromatography. Antimicrobial activities were evaluated by the capacity to inhibit different bacterial strains by absorbance measurement (OD 600 nm). Antimicrobial activity was expressed as IC50 [108]. Another study demonstrated that extracts from *Saccharina longicruris* also had effectiveness against methicillin-resistant *Staphylococcus aureus*. In this research, the alga proteins were extracted from commercially powdered forms and hydrolyzed with the enzyme trypsin in order to recover antibacterial peptides. Protein extract of both fractions was determined by liquid growth inhibition assays using an optimized microdilution method at algae concentrations from 0.31 mg/mL to 2.5 mg/mL [109,110]. 

Moreover, bioactive compounds isolated from the green macroalga *Ulva armoricana* inhibit the growth of both Gram-positive and Gram-negative bacteria. In this evaluation, *Ulva armoricana* algae were collected, freeze-dried, and ground into powder to obtain marine-sulfated polysaccharide (MSP). The antimicrobial activity was tested at concentrations from 50 to 0.04 mg/mL^−1^ on MH agar or MH with 5% horse serum. A bacterial suspension (10⁴ CFU, 2–5 μL) was inoculated onto agar plates with increasing MSP concentrations and incubated at 37 °C for 24 h. The bacterial sensitivity was recorded, and the minimal inhibitory concentration was determined after 24 h. The results highlighted that the most sensitive pathogens to exposure of *Ulva armoricana* were *Pasteurella multocida*, *Staphylococcus aureus*, *Mannheimia haemolytica*, *Erysipelothrix rhusiopathiae*, *Streptococcus*, and *Enterococcus cecorum* [111]. These pathogens cause conditions such as reproductive disorders, pneumonia, and diarrhea that lead to high mortality in livestock and significant economic losses [112,113]. Furthermore, recent studies on the brown (*Ascophyllum nodosum*, AN) and green alga (*Ulva lactuca*, UL) have revealed that their extracts significantly inhibited the growth of *Escherichia coli* strains. Commercially purchased seaweed biomass of the tested species was extracted using ethanol as a solvent. The bacteria were supplemented with different concentrations (0, 1.44, 2.87, 5.75, 11.50, and 23.0 mg/mL) of seaweed extracts, and growth was monitored by absorbance measurement (OD620). Thus, AN and UL seaweed extracts reveal promising antibacterial effects [114]. The literature results provide a new insight into the development of natural antimicrobial agents applicable against infectious pathogens in livestock farming [101]. 

Microalgae and macroalgae have the potential to serve as biodegradable antibiotic agents to address antimicrobial resistance [102]. However, it should be noted that further research on algal extract is required to fully understand their potential and how they can be used to fight bacterial infections [115]. The development of new antibiotics is a complex and time-consuming process that requires rigorous testing and clinical trials to ensure their safety and efficacy [116]. Thus, while algae extracts hold potential as an antibiotic, alternative further research is needed to fully understand their potential and how they can be used to combat bacterial infections. Rigorous clinical trials will be essential to validate their effectiveness [101,102].

**Table 3:** Reported antibacterial activity of algae extracts.

Algae Species	Bioactive Agent	Experimental Conditions	Extraction Method and Concentration	Antimicrobial Activity	Reference
*Haematococcus pluvialis*	Astaxanthin from red phase (pigments).	Culture was grown in Bold’s Basal Medium with NaNO_3_ (0.75 g/L). Green phase was cultured in 20 L Carboys bubbled at 25 °C in light/dark cycles (16:8 h). Red phases were transferred to nitrogen-deprived medium and illuminated with 200 µmol m^−2^ s^−1^ for 6 days.	Hexane and ethanol as solvents were used at 50, 100, 150, and 200 °C for 20 min. Pressurized liquid extraction was applied by freezing and mashing with liquid nitrogen in a ceramic mortar. The pathogen was supplemented in concentration of 1 mg/mL.	Exhibited bactericidal action against *Escherichia coli*, *Staphylococcus aureus*, *Escherichia coli*, *Candida albicans*, *Aspergillus niger* from ethanol extract.	[102,117]
*Chlorella* *vulgaris*	Flavonoids, tannins, phenolic compounds, terpenoids.	Alga was collected from surface lake water bodies. Pure cultures prior to the stationary phase of growth (10 days) were then collected by centrifuging (10,000 rpm, 3 min). The pellets were shade-dried and ground into a coarse powder with a mechanical grinder.	The dried powders (20 gm) were extracted using serial with chloroform, acetone, ethanol solvents (10 mL of solvent in 1 gm of powder). The pathogen was supplemented at concentration of 100 mg/mL.	Extracts prepared with different solvents suppressed the growth of *E.coli, Klebsilla* sp., *Bacillus* sp., and *Pseudomonas* sp.	[118]
*Isochrysis* *galbana*	Myristic acid, behenic acid, oleic acid, tearidonic acid (lipids).	Three different strains were maintained as monospecific, nonaxenic batch cultures in 500 mL Erlenmeyer flasks with 200 mL “f” medium. The initial culture concentration was 1 × 10^5^ cells mL^−1^. The conditions were 22 ± 1 °C, salinity 33 ± 1‰, 24 h of continuous light at 110 μmol photons m^−2^ s^−1^.	No extraction method was used. The bacteria flasks were inoculated with alga at 1 × 10^5^ cells mL^−1^.	Isochrysis galbana inhibits the growth of *Vibrio alginolyticus*, *Vibrio campbellii*, and *Vibrio harveyi*.	[101,119]
*Dunaliella* *salina*	β-cyclocitral and α- and β-ionone (carotenoid derivatives), 2-hexadiene-1-ol,3,7,11,15-tetra-methyl (neophytadiene, phytol), palmitic, α-linolenic, and oleic acids (fatty acids).	Freeze-dried microalgae were stored under dry and dark conditions.	Extraction in solvents (hexane, petroleum ether, ethanol, and water) and different temperatures (40, 100, and 160 °C) in 15 min as extraction time were used by pressurized liquid extracts. In analysis of volatile fractions, petroleum ether and hexane extracts were injected at concentration of 2 mg/mL, ethanol extracts at concentration of 25 mg/mL.	Extracts suppressed the growth of *Escherichia coli*, *Staphylococcus aureus*, *Candida albicans*, and *Aspergillus niger*. The best antimicrobial activity was obtained at highest extraction temperature in ethanol solvent.	[120]
*Scenedesmus obliquus*	Palmitic acid, linoleic acid (fatty acids).	Batch cultures were grown (30 days) in flasks containing 200 mL of medium, at 25 °C, and under continuous illumination with fluorescent daylight lamps (35 µmol_photon_ m^−2^ s^−1^) using OHM and TAP as culture media.	The 30-day cultures were centrifuged (1790× *g*, 10 min, 15 °C), and pellet was homogenized in 25 mL of ethanol–water (1:1), the cells were then centrifugated. Intra- and extracellular extracts were used. A 500 µL aliquot was added to 9.5 mL of medium, along with 100 µL of each pathogen suspension.	Extracts of *Scenedesmus obliquus* exhibited antibacterial activity against *Pseudomonas aeruginosa*, *Escherichia coli Staphylococcus* *aureus*.	[121]
*Phaeodactylum* *tricornutum*	Eicosapentaenoic acid (fatty acids).	Strain was maintained at +4 °C on 2216E medium, Luria–Bertani (LB), nutrient, or YPD agar.	The compound was extracted from a cell pellet in 50 mL methanol/water (5:1) for 16 h on ice. After centrifugation (5525× *g*, 1 h, 4 °C), the extract was dried (~4 h, 30 °C) and reconstituted in 20 mL of 70% methanol. Aliquots of 75 μL were tested.	A fatty acid isolated from *Phaeodactylum tricornutum* exhibited antibacterial activity against multi-resistant *Staphylococcus aureus*.	[122]
*Tetrasekmis suecica*	Methyl carprate, Methyl stearate, Decoic acid, Palmitic acid, Nonoic acid, and Caprylic acid (fatty acids).	Filtered seawater was placed in a 250 mL flask, amended with Miquell’s medium, and autoclaved. After sterilization, 10% of actively growing inoculum was added into culture flasks and was incubated at 28 ± 2 °C under 1000 lux light for 8 days. Once the culture reached its maximum exponential phase, light intensity was reduced.	Algal cells were centrifuged (200 rpm, 10 min); the pellet was air-dried. Dried cells (10 g) were extracted in 100 mL of organic solvents (acetone, n-butanol, isopropanol, acetone + n-butanol (1:1), acetone + isopropanol (1:1), acetone + chloroform (1:1), butanol + isopropanol (1:1), chloroform + methanol (1:1)) under stirring (50 rpm) for 7 d. The solution was filtered and dried in a desiccator (40 °C, 24 h). The dried powder was dissolved to obtain a 50 mg/mL extract.	Chloroform + methanol (1:1) extract of *Tetraselmis suecica* exhibited antibacterial activity against *Proteus* sp. and *Streptococcus pyogens.*	[123]

### 4.2. Mode of Action of the Antimicrobial Properties of Algae

The antimicrobial properties of algae are linked to the presence of biologically active compounds. These bioactive compounds possess specific chemical structures that allow them to function as beneficial substances, leading to the inhibition of harmful bacterial pathogens. Algae are a source of biologically active compounds, including polyphenols, polysaccharides, proteins and peptides, lipids, and pigments [124]. The antimicrobial action of polyphenols is linked to their ability to alter membrane permeability causing cell lysis, inhibiting enzymes and various metabolic pathways, binding to surface molecules, and other mechanisms. This activity appears to be related to the number of hydroxyl groups and also the degree of polymerization [115]. The antimicrobial properties of polysaccharides from algae are attributed to their interaction with glyco-receptors of the bacterial cell wall, membrane components and nucleic acids and polysaccharides. Those interactions disrupt the membrane stability and cellular functions [103]. Various factors influence this activity, including molecular weight, charge density, structure, and conformation [3]. The inhibitory effects of proteins and peptides from algae are associated with their amphiphilic nature, enabling them to interact with both polar and non-polar sites of bacterial membranes. This interaction results in the formation of pores, disrupting the membrane and causing cellular rupture. However, in many cases, the exact mechanism of action remains unclear [3]. The antimicrobial activity of algal lipids and fatty acids from algae has been attributed to their ability to inhibit the electron transport chain and oxidative phosphorylation in cell membranes. This leads to the formation of peroxidation and auto-oxidation degradation products, ultimately causing cellular lysis [103]. The antimicrobial mechanism of pigments has not been fully elucidated. Among the most studied pigments are the carotenoids, which act by accumulating lysozyme, an enzyme capable of digesting bacterial cell walls [125]. Another carotenoid, the proposed mode of action of fucoxanthin includes an increase in permeability, cytoplasm leakage, and inhibition of nucleic acid formation [103,126].

In summary, algae have shown great potential as therapeutic agents due to their various bioactive compounds. However, explaining the mode of action of the antimicrobial activity of algae is challenging because they are typically assessed as extracts rather than as individual compounds composed of different biomolecules. Therefore, the antimicrobial effect is likely due to a synergistic interaction between these compounds [3]. Thus, the exact mechanism of action of the antimicrobial properties of algae is still not fully understood and requires further research [110,111,127]. In order to realize the potential of algal ingredients to be translated into pharmaceutical agents, of key importance will also be to validate activities observed in vitro into in vivo disease models and clinical research studies.

## 5. Significance of Swine Animal Models for the Validation of Bioactivities of Algal Ingredients and Importance of Algal Therapeutic Application in Gut Immunity 

Animal models are useful for understanding disease progression, discovering and validating therapeutic drugs, and assessing pharmacodynamics and potential toxic side effects [128,129]. A good animal research model must be standardized, have reproducibility, cross-study validity, and bidirectional translation between species, and allow different levels of research in order to reach reliable and useful conclusions [128]. 

Classically, rodents have been the preferred model for research due to them being easy to handle, requiring minimal space to maintain, and the ability to be bred to produce large numbers in a relatively short time frame. However, rodents exhibit many anatomical and physiological differences with humans that can limit their applicability. Further, several rodent models do not fully recapitulate the human disease [130]. Swine as a model is relatively new for studying human health and disease status compared to rodents. However, due to their similarity to humans in terms of anatomic, physiologic, metabolomics, pathophysiological, and genetic features and similarity to human size, pigs have greatly advanced in understanding human conditions, establishing them as an excellent choice for an animal model rather than rodents. Moreover, recent advances in genetic engineering of the swine genome enhance their utility as models of human genetic diseases [130,131]. Besides, the anatomical structure of the pig gastrointestinal tract, particularly the small intestine and stomach, and the composition of the intestinal microflora, are analogous to the structures in humans [132]. Consequently, primary intestinal functions such as water and nutrient absorption and microbial fermentation are comparable to those in the human gastrointestinal tract [133]. Further, the swine metabolism closely resembles the human metabolism, providing a distinct advantage over rodents. Pigs are particularly suitable for investigating drug disposition because they share a significant number of membrane transport and enzymatic proteins with humans [133].

Additionally, several immune cells and processes of the adaptive and innate immune systems, along with the recognition of innate immunity activators by macrophages, are comparable to those in humans, making pigs an excellent model for studying intestinal inflammation. Pigs also have an abundance of intraepithelial cells compared to mice and humans, making them an effective model for understanding how these cells rapidly respond to bacterial antigens in the intestine [133]. Thus, pigs are also susceptible to many diseases that occur in humans, such as diabetes, heart disease, obesity, and neurodegenerative diseases [128,134]. In addition, due to their large size and long lifespan, pigs are a convenient model for testing the long-term effects of drugs and other treatments. 

Consequently, swine fulfill regulations of good animal research models and may be commonly used in human disease research. Due to their similarity to humans, they are useful in biomedical research, such as studies on metabolic, cardiologic, neurological, and immunological diseases [135,136]. Therefore, studies in a pig model can provide valuable information on the pathogenesis and treatment of diseases in humans [129,136].

Algae extracts with their natural origin have diverse therapeutic applications, including human diseases. Antioxidant, anti-inflammatory, antibacterial, and antiviral properties, as well as their ability to reduce the risk of metabolic, cardiac, neurological, and immune diseases, attract the attention of scientists and researchers [137,138]. In clinical trials, algae extracts are often used in the form of dietary supplements. Research on pig models, in particular, can help determine the effect of algae extracts on selected human diseases, such as metabolic diseases, heart diseases, neurological diseases, and diseases of the immune system. By using a pig model, researchers can test the effectiveness and safety of algae extracts as potential therapies for humans. Ultimately, research in a pig model may help to understand the mechanisms of action of algae extracts and how they can help treat diseases in humans. Along this line, previous research revealed that algal β-glucan improved gut health and immune responses in *Escherichia coli*-infected weaned pigs [139]. In the context of combating anemia, another study established that supplementation with two types of defatted green microalgae (*Nannochloropsis oceanica* and *Desmodesmus* sp.) alleviated anemia in a weanling pig model by providing a dual dietary source of both highly bioavailable iron and protein [140]. Other research also demonstrated promising effects of laminarin derived from the brown seaweed *Laminaria digitate* in pigs exposed to pro-inflammatory LPS challenge [141]. In particular, dietary supplementation with laminarin led to increased expression of the protective gut mucin genes (MUC2 and MUC4) [141].

Moreover, the origin of numerous prevalent chronic global disorders such as metabolomics syndrome, inflammatory disease, immunodeficiency, and cancer has been associated with dysbiosis in gut microbiota [142]. Numerous studies have demonstrated that consuming seaweed components may beneficially modulate the microbiota of the mammalian gut [143]. Marine algae polysaccharides can enhance the activities of beneficial bacteria populations and stimulate the production of functional metabolites by gut microbiota. These polysaccharides are crucial for maintaining the barrier function of the intestines, regulating epithelial proliferation, and modulating immune responses [142]. Polysaccharides isolated from marine algae play a significant role in regulating intestinal microbiota, thereby benefiting host health [144] and positively manipulating the gut microbiome [145]. Seaweed polysaccharides have a pleiotropic effect, enabling them to influence therapeutic targets for inflammatory bowel disease, including inflammatory cytokines, adhesion molecules, intestinal microbiota, and intestinal epithelial cells [146]. Furthermore, fatty acids derived from seaweeds have been demonstrated to modulate cell signaling pathways involved in metabolic homeostasis and immune responses [145]. Likewise, the functional properties of macroalgal glycans influence gut microbiota and immune cells, potentially leading to the development of targeted nutraceutical products [147].

Thereby, algae and algae-derived compounds have been proven to positively influence gut immunity and modulate intestinal microbiota in the prevention of various diseases. Maintaining a healthy gut is a significant area of research in humans, with the gut often referred to as the second brain. Thus, preserving or enhancing gut health is crucial for a robust immune system and overall well-being. Dietary approaches, particularly the use of various plant-derived nutraceuticals, may be the most effective method for sustaining a healthy gut microbiota population [142].

## 6. Future Research Directions

While the current literature reviewed in this work highlights the promising bioactive potential of algae-derived compounds in combating inflammation, oxidative stress, and bacterial infections, further research is needed to elucidate more details of their mechanisms of action and patterns of in vivo action. In particular, more rigorous preclinical studies using animal models like pigs, due to their physiological similarity to humans, can help confirm the therapeutic effects observed in vitro. Additionally, future research should focus on identifying the specific molecular pathways modulated by various algal compounds, optimizing extraction and formulation techniques to maximize efficacy, and assessing the long-term safety of these compounds in clinical settings. Finally, integrating advanced omics technologies could provide deeper insights into the interactions between algal bioactives and host microbiota, opening new avenues for personalized medicine and nutraceutical applications.

## 7. Conclusions

The conducted literature review indicates that algae extract and isolated algae ingredients show promise in the management of various human diseases, including cancer, diabetes, and neurodegenerative conditions. Importantly, many of these diseases are associated with chronic inflammation, and algae and algae-derived ingredients might offer benefits through their anti-inflammatory and antioxidant effect properties, mitigating oxidative stress associated with inflammatory conditions. In particular, fucoxanthin has demonstrated strong anti-inflammatory and antioxidant effects in multiple research works, making it a promising candidate for future study of its therapeutic potential. Additionally, polysaccharides such as fucoidan have shown promise in modulating immune responses by enhancing macrophage activity and inducing apoptosis in tumor cells, which might be of special interest in the context of cancer immunotherapy. Macrophages play a pivotal role in regulating inflammatory responses, and multiple algal extracts and isolated ingredients have demonstrated their potential to influence inflammation through macrophage-specific effects. These effects include the suppression of pro-inflammatory cytokines, antioxidative effects, the regulation of cell fate, and modulation of key signaling pathways such as the activation of MAPK/NF-κB, modulation of P13K/AKT- and JAK/STAT-signaling, and the activation of Nrf2-mediated antioxidant defense mechanisms. Further, algal extracts and isolated ingredients have also demonstrated promise in the realm of antimicrobial activities, which could potentially lead to the development of new antimicrobial therapies for medical use and serve as an antibiotic replacement for use in farm animals. In order to further develop algal ingredients exhibiting promising in vitro effects for use in patients or in farming, rigorous animal validation research is of key importance, and pigs in particular represent an efficient and promising laboratory model for the study of algal bioactive compounds. Consequently, research conducted up to now indicates the special promise of algae extracts in counteracting diseases by reducing inflammation and oxidative stress and regulating apoptosis. Thus, algae extracts and isolated compounds may have significant therapeutic potential in the management of human and animal diseases as well as in improving gut immunity, and further research is needed to investigate their safety and efficacy in preclinical animal research models and in human clinical trials. Future studies should especially prioritize clinical trials to evaluate the bioactivities of relevant compounds in vivo, for example, focusing on constituents such as astaxanthin, the efficacy of which has already shown promise in numerous preclinical models.

## Figures and Tables

**Table 2:** Bioactive constituents found in algae with the ability to modulate MAPK/NF-κB pathways in LPS-induced macrophages while maintaining low cytotoxicity.

Bioactive Ingredients	Algae Species	Bioactive Agent	Effect of the Bioactive Compounds	Cytotoxicity	Reference
Microalgae					
Lipids/pigments	*Nostoc commune* var. *sphaeroides*	Free fatty acids, triacylglycerol, wax esters/steryl esters, and moreover pigments.	Inhibition NF-κB DNA binding activity.	Non-cytotoxic in the tested range (25–200 μg/mL).	[23,29]
	*Dunaliella salina*	Carotenoids (all-trans forms of α-carotene, β-carotene, lutein, and zeaxanthin, 13- or 13′-cis-β-carotene, 9- or 9′-cis-α-carotene, and 9- or 9′-cis-β-carotene).	Reduction of IkBα phosphorylation. Reduction of p50 but not p65 translocations.	Cytotoxic effect above 50 μM.	[55]
	*Haematoccus pluvialis*	Carotenoids (astaxanthin).	Reduction of p65 translocation to the nucleus.	-	[46]
Polysaccharide	*Coccomyxa* *gloeobotrydiform*	Not mentioned.	Inhibition of NF-κB p65 phosphorylation, inhibition of phosphorylation of MAPK molecules including p38, JNK, and ERK1/2.	Non-cytotoxic in the tested range (2 or 4 mg/mL as determined by LDH release assay).	[4]
Macroalgae					
Lipids/pigments	*Porphyra dioica* *Palmaria palmata* *Chondrus crispus* *Pavlova lutheri* *(microalga)*	n-3 PUFA and pigments (chlorophyll a, β-carotene).	Downregulation of the expression of 14 pro-inflammatory genes (TLR1, TLR2, TLR4, TLR8, TRAF5, TRAF6, TNFSF18, IL6R, IL23, CCR1, CCR4, CCL17, STAT3, MAP3K1) indicating inhibition of the signaling pathways mediated via toll-like receptors, chemokines, and NF-κB.	Non-cytotoxic in the tested range.	[40]
Phenols	*Ecklonia cava*	Phloroglucinol derivatives.	Inhibition of expression of phosphorylated proteins JNK, ERK, and p38.	Cytotoxic effect above 50 μM.	[43]
	*Ecklonia stolonifera*	Phlorotannins (phlorofucofuroeckol A).	Inhibition of JNK phosphorylation. Inhibition of AP-1 activity. Inhibition of expression of Fas-mediated apoptotic proteins including Fas ligand, cleaved caspase-8, cleaved caspase-3.	-	[28,56]
Polysaccharide	*Gelidium crinale*	Glycans (sulfated polysaccharides).	Inhibition of p38, JNN, and ERK phosphorylation. Inhibition of p65 and IkBα translocation.	Non-cytotoxic in the tested range (1–1000 μg/mL).	[57]
	*Ascophyllum nodosum*	Ascophyllan (sulfated polysaccharide).	Increased nuclear translocation of p65 and phosphorylation and degradation of IκB-α. Increase in the phosphorylated levels of ERK, p38, and JNK.	No significant cytotoxic effects up to 1000 μg/mL.	[50]
	*Sargassum horneri*	Fucoidan (sulfated polysaccharide).	Inhibition of p50 and p65 translocation. Inhibition of p38 and ERK activation.	Non-cytotoxicity in the range of 12.5 μg to 50.0 μg.	[58]
	*Saccharina japonica*	Fucoidan (sulfated polysaccharide).	Inhibition of phosphorylation and proteolytic degradation of cytoplasmic IKK-α, IκB-α, p50, and p65. Suppression of ERK1/2, JNK, and p38 MAP phosphorylation.	Non-cytotoxicity in the range of up to 200 μg/mL.	[7]

## Data Availability

Not applicable.

## References

[1] Kholssi R., Lougraimzi H., Grina F., Lorentz J.F., Silva I., Castaño-Sánchez O., Marks E.A.N. (2022). Green Agriculture: A Review of the Application of Micro- and Macroalgae and Their Impact on Crop Production on Soil Quality. J. Soil Sci. Plant Nutr..

[2] Tan P.X., Thiyagarasaiyar K., Tan C.-Y., Jeon Y.-J., Nadzir M.S.M., Wu Y.-J., Low L.-E., Atanasov A.G., Ming L.C., Liew K.B. (2021). Algae-Derived Anti-Inflammatory Compounds against Particulate Matters-Induced Respiratory Diseases: A Systematic Review. Mar. Drugs.

[3] Silva A., Silva S.A., Carpena M., Garcia-Oliveira P., Gullón P., Barroso M.F., Prieto M.A., Simal-Gandara J. (2020). Macroalgae as a Source of Valuable Antimicrobial Compounds: Extraction and Applications. Antibiotics.

[4] Dai B., Wei D., Zheng N., Chi Z., Xin N., Ma T., Zheng L., Sumi R., Sun L. (2018). Coccomyxa Gloeobotrydiformis Polysaccharide Inhibits Lipopolysaccharide-Induced Inflammation in RAW 264.7 Macrophages. Cell. Physiol. Biochem..

[5] Daskalaki M., Vyrla D., Harizani M., Doxaki C., Eliopoulos A., Roussis V., Ioannou E., Tsatsanis C., Kampranis S. (2019). Neorogioltriol and Related Diterpenes from the Red Alga *Laurencia* Inhibit Inflammatory Bowel Disease in Mice by Suppressing M1 and Promoting M2-Like Macrophage Responses. Mar. Drugs.

[6] Kim A.-R., Lee M.-S., Shin T.-S., Hua H., Jang B.-C., Choi J.-S., Byun D.-S., Utsuki T., Ingram D., Kim H.-R. (2011). Phlorofucofuroeckol A Inhibits the LPS-Stimulated INOS and COX-2 Expressions in Macrophages via Inhibition of NF-ΚB, Akt, and P38 MAPK. Toxicol. In Vitro.

[7] Ye J., Chen D., Ye Z., Huang Y., Zhang N., Lui E.M.K., Xue C., Xiao M. (2020). Fucoidan Isolated from *Saccharina japonica* Inhibits LPS-Induced Inflammation in Macrophages via Blocking NF-ΚB, MAPK and JAK-STAT Pathways. Mar. Drugs.

[8] Li C.-Q., Ma Q.-Y., Gao X.-Z., Wang X., Zhang B.-L. (2021). Research Progress in Anti-Inflammatory Bioactive Substances Derived from Marine Microorganisms, Sponges, Algae, and Corals. Mar. Drugs.

[9] Tabarzad M., Atabaki V., Hosseinabadi T. (2020). Anti-Inflammatory Activity of Bioactive Compounds from Microalgae and Cyanobacteria by Focusing on the Mechanisms of Action. Mol. Biol. Rep..

[10] Rocha D.H.A., Pinto D.C.G.A., Silva A.M.S. (2022). Macroalgae Specialized Metabolites: Evidence for Their Anti-Inflammatory Health Benefits. Mar. Drugs.

[11] Ghosh S., Sarkar T., Pati S., Kari Z.A., Edinur H.A., Chakraborty R. (2022). Novel Bioactive Compounds from Marine Sources as a Tool for Functional Food Development. Front. Mar. Sci..

[12] Sagnia B., Fedeli D., Casetti R., Montesano C., Falcioni G., Colizzi V. (2014). Antioxidant and Anti-Inflammatory Activities of Extracts from *Cassia alata*, *Eleusine indica*, *Eremomastax speciosa*, *Carica papaya* and *Polyscias fulva* Medicinal Plants Collected in Cameroon. PLoS ONE.

[13] Mhadhebi L., Mhadhebi A., Robert J., Bouraoui A. (2014). Antioxidant, Anti-Inflammatory and Antiproliferative Effects of Aqueous Extracts of Three Mediterranean Brown Seaweeds of the Genus *Cystoseira*. Iran J. Pharm. Res..

[14] Fernando I.P.S., Nah J.-W., Jeon Y.-J. (2016). Potential Anti-Inflammatory Natural Products from Marine Algae. Environ. Toxicol. Pharmacol..

[15] Obluchinskaya E.D., Pozharitskaya O.N., Shikov A.N. (2022). In Vitro Anti-Inflammatory Activities of Fucoidans from Five Species of Brown Seaweeds. Mar. Drugs.

[16] Hernández-Ledesma B., Hsieh C.-C., de Lumen B.O. (2009). Antioxidant and Anti-Inflammatory Properties of Cancer Preventive Peptide Lunasin in RAW 264.7 Macrophages. Biochem. Biophys. Res. Commun..

[17] Swanson K.V., Deng M., Ting J.P.-Y. (2019). The NLRP3 Inflammasome: Molecular Activation and Regulation to Therapeutics. Nat. Rev. Immunol..

[18] Remya R.R., Samrot A.V., Kumar S.S., Mohanavel V., Karthick A., Chinnaiyan V.K., Umapathy D., Muhibbullah M. (2022). Bioactive Potential of Brown Algae. Adsorpt. Sci. Technol..

[19] Pangestuti R., Vo T.-S., Ngo D.-H., Kim S.-K. (2013). Fucoxanthin Ameliorates Inflammation and Oxidative Reponses in Microglia. J. Agric. Food Chem..

[20] Chae D., Manzoor Z., Kim S., Kim S., Oh T.-H., Yoo E.-S., Kang H.-K., Hyun J.-W., Lee N., Ko M.-H. (2013). Apo-9′-Fucoxanthinone, Isolated from *Sargassum muticum*, Inhibits CpG-Induced Inflammatory Response by Attenuating the Mitogen-Activated Protein Kinase Pathway. Mar. Drugs.

[21] Mease P.J., Armstrong A.W. (2014). Managing Patients with Psoriatic Disease: The Diagnosis and Pharmacologic Treatment of Psoriatic Arthritis in Patients with Psoriasis. Drugs.

[22] Doan T., Massarotti E. (2005). Rheumatoid Arthritis: An Overview of New and Emerging Therapies. J. Clin. Pharmacol..

[23] Park Y.-K., Rasmussen H.E., Ehlers S.J., Blobaum K.R., Lu F., Schlegal V.L., Carr T.P., Lee J.-Y. (2008). Repression of Proinflammatory Gene Expression by Lipid Extract of Nostoc Commune Var Sphaeroides Kützing, a Blue-Green Alga, via Inhibition of Nuclear Factor-ΚB in RAW 264.7 Macrophages. Nutr. Res..

[24] Burayk S., Oh-hashi K., Kandeel M. (2022). Drug Discovery of New Anti-Inflammatory Compounds by Targeting Cyclooxygenases. Pharmaceuticals.

[25] Atanasov A.G., Zotchev S.B., Dirsch V.M., Supuran C.T. (2021). Natural Products in Drug Discovery: Advances and Opportunities. Nat. Rev. Drug Discov..

[26] Tzvetkov N.T., Kirilov K., Matin M., Atanasov A.G. (2023). Natural Product Drug Discovery and Drug Design: Two Approaches Shaping New Pharmaceutical Development. Nephrol. Dial. Transplant..

[27] Schmid-Schönbein G.W. (2006). Analysis of Inflammation. Annu. Rev. Biomed. Eng..

[28] Kim E.J., Park S.Y., Lee J.-Y., Park J.H.Y. (2010). Fucoidan Present in Brown Algae Induces Apoptosis of Human Colon Cancer Cells. BMC Gastroenterol..

[29] Ku C.S., Pham T.X., Park Y., Kim B., Shin M.S., Kang I., Lee J. (2013). Edible Blue-Green Algae Reduce the Production of pro-Inflammatory Cytokines by Inhibiting NF-ΚB Pathway in Macrophages and Splenocytes. Biochim. Biophys. Acta (BBA) Gen. Subj..

[30] Maj D., Migdał Ł., Zapletal P. (2023). Effects of Dietary Supplementation with Algae, Sunflower Oil, or Soybean Oil, and Age on Fat Content, Fatty Acid Profile and the Expression of Related Genes in Rabbits. Anim. Sci. Pap. Rep..

[31] Jayawardena T.U., Sanjeewa K.K.A., Lee H.-G., Nagahawatta D.P., Yang H.-W., Kang M.-C., Jeon Y.-J. (2020). Particulate Matter-Induced Inflammation/Oxidative Stress in Macrophages: Fucosterol from *Padina boryana* as a Potent Protector, Activated via NF-ΚB/MAPK Pathways and Nrf2/HO-1 Involvement. Mar. Drugs.

[32] Sanjeewa K.K.A., Fernando I.P.S., Kim H.-S., Jayawardena T.U., Ryu B., Yang H.-W., Ahn G., Lee W., Jeon Y.-J. (2020). Dieckol: An Algal Polyphenol Attenuates Urban Fine Dust-Induced Inflammation in RAW 264.7 Cells via the Activation of Anti-Inflammatory and Antioxidant Signaling Pathways. J. Appl. Phycol..

[33] Watkins L.R., Maier S.F., Goehler L.E. (1995). Immune Activation: The Role of pro-Inflammatory Cytokines in Inflammation, Illness Responses and Pathological Pain States. Pain.

[34] Mhadhebi L. (2011). Anti-Inflammatory, Anti-Proliferative and Anti-Oxidant Activities of Organic Extracts from the Mediterranean Seaweed, *Cystoseira crinita*. Afr. J. Biotechnol..

[35] Mhadhebi L., Dellai A., Clary-Laroche A., Said R.B., Robert J., Bouraoui A. (2012). Anti-Inflammatory and Antiproliferative Activities of Organic Fractions from the Mediterranean Brown Seaweed, *Cystoseira compressa*. Drug Dev. Res..

[36] Harada H., Kamei Y. (1997). Selective Cytotoxicity of Marine Algae Extracts to Several Human Leukemic Cell Lines. Cytotechnology.

[37] Zhang Z., Teruya K., Yoshida T., Eto H., Shirahata S. (2013). Fucoidan Extract Enhances the Anti-Cancer Activity of Chemotherapeutic Agents in MDA-MB-231 and MCF-7 Breast Cancer Cells. Mar. Drugs.

[38] Álvarez-Gómez F., Korbee N., Casas-Arrojo V., Abdala-Díaz R.T., Figueroa F.L. (2019). UV Photoprotection, Cytotoxicity and Immunology Capacity of Red Algae Extracts. Molecules.

[39] Choi Y.K., Ye B.-R., Kim E.-A., Kim J., Kim M.-S., Lee W.W., Ahn G.-N., Kang N., Jung W.-K., Heo S.-J. (2018). Bis (3-Bromo-4,5-Dihydroxybenzyl) Ether, a Novel Bromophenol from the Marine Red Alga Polysiphonia Morrowii That Suppresses LPS-Induced Inflammatory Response by Inhibiting ROS-Mediated ERK Signaling Pathway in RAW 264.7 Macrophages. Biomed. Pharmacother..

[40] Robertson R., Guihéneuf F., Bahar B., Schmid M., Stengel D., Fitzgerald G., Ross R., Stanton C. (2015). The Anti-Inflammatory Effect of Algae-Derived Lipid Extracts on Lipopolysaccharide (LPS)-Stimulated Human THP-1 Macrophages. Mar. Drugs.

[41] Chakraborty K., Thambi A., Dhara S. (2023). Sulfated Polygalactofucan from Triangular Sea Bell *Turbinaria* Decurrens Attenuates Inflammatory Cytokines on THP-1 Human Monocytic Macrophages. Int. J. Biol. Macromol..

[42] Amin A.R., Attur M., Abramson S.B. (1999). Nitric Oxide Synthase and Cyclooxygenases. Curr. Opin. Rheumatol..

[43] Kong C.-S., Kim J.-A., Ahn B.-N., Kim S.-K. (2011). Potential Effect of Phloroglucinol Derivatives from *Ecklonia cava* on Matrix Metalloproteinase Expression and the Inflammatory Profile in Lipopolysaccharide-Stimulated Human THP-1 Macrophages. Fish Sci..

[44] Banskota A.H., Stefanova R., Sperker S., Lall S.P., Craigie J.S., Hafting J.T., Critchley A.T. (2014). Polar Lipids from the Marine Macroalga Palmaria Palmata Inhibit Lipopolysaccharide-Induced Nitric Oxide Production in RAW264.7 Macrophage Cells. Phytochemistry.

[45] Takahashi S., Sakamaki M., Ferdousi F., Yoshida M., Demura M., Watanabe M.M., Isoda H. (2018). Ethanol Extract of *Aurantiochytrium mangrovei* 18W-13a Strain Possesses Anti-Inflammatory Effects on Murine Macrophage RAW264 Cells. Front. Physiol..

[46] Farruggia C., Kim M.-B., Bae M., Lee Y., Pham T.X., Yang Y., Han M.J., Park Y.-K., Lee J.-Y. (2018). Astaxanthin Exerts Anti-Inflammatory and Antioxidant Effects in Macrophages in NRF2-Dependent and Independent Manners. J. Nutr. Biochem..

[47] McCauley J.I., Meyer B.J., Winberg P.C., Ranson M., Skropeta D. (2015). Selecting Australian Marine Macroalgae Based on the Fatty Acid Composition and Anti-Inflammatory Activity. J. Appl. Phycol..

[48] Zhang R., Chen J., Mao X., Qi P., Zhang X. (2019). Anti-Inflammatory and Anti-Aging Evaluation of Pigment–Protein Complex Extracted from *Chlorella pyrenoidosa*. Mar. Drugs.

[49] Yi L., Wang Q., Luo H., Lei D., Tang Z., Lei S., Xiao H. (2022). Inhibitory Effects of Polyphenols-Rich Components from Three Edible Seaweeds on Inflammation and Colon Cancer in Vitro. Front. Nutr..

[50] Jiang Z., Okimura T., Yamaguchi K., Oda T. (2011). The Potent Activity of Sulfated Polysaccharide, Ascophyllan, Isolated from Ascophyllum Nodosum to Induce Nitric Oxide and Cytokine Production from Mouse Macrophage RAW264.7 Cells: Comparison between Ascophyllan and Fucoidan. Nitric Oxide.

[51] Kim K.-N., Heo S.-J., Yoon W.-J., Kang S.-M., Ahn G., Yi T.-H., Jeon Y.-J. (2010). Fucoxanthin Inhibits the Inflammatory Response by Suppressing the Activation of NF-ΚB and MAPKs in Lipopolysaccharide-Induced RAW 264.7 Macrophages. Eur. J. Pharmacol..

[52] Muxel S.M., Laranjeira-Silva M.F., Carvalho-Sousa C.E., Floeter-Winter L.M., Markus R.P. (2016). The RelA/cRel nuclear factor-κB (NF-κB) dimer, crucial for inflammation resolution, mediates the transcription of the key enzyme in melatonin synthesis in RAW 264.7 macrophages. J. Pineal Res..

[53] Yao W., Qiu H.-M., Cheong K.-L., Zhong S. (2022). Advances in Anti-Cancer Effects and Underlying Mechanisms of Marine Algae Polysaccharides. Int. J. Biol. Macromol..

[54] Bittner M., Štern A., Smutná M., Hilscherová K., Žegura B. (2021). Cytotoxic and Genotoxic Effects of Cyanobacterial and Algal Extracts—Microcystin and Retinoic Acid Content. Toxins.

[55] Yang D.-J., Lin J.-T., Chen Y.-C., Liu S.-C., Lu F.-J., Chang T.-J., Wang M., Lin H.-W., Chang Y.-Y. (2013). Suppressive Effect of Carotenoid Extract of *Dunaliella salina* Alga on Production of LPS-Stimulated pro-Inflammatory Mediators in RAW264.7 Cells via NF-ΚB and JNK Inactivation. J. Funct. Foods.

[56] Lee M.-S., Kim J.-I., Utsuki T., Park N.-G., Kim H.-R. (2012). Cytoprotective Effects of Phlorofucofuroeckol A Isolated from *Ecklonia stolonifera* against Tacrine-Treated HepG2 Cells. Fitoterapia.

[57] Pei Y., Yang S., Xiao Z., Zhou C., Hong P., Qian Z.-J. (2021). Structural Characterization of Sulfated Polysaccharide Isolated from Red Algae (*Gelidium crinale*) and Antioxidant and Anti-Inflammatory Effects in Macrophage Cells. Front. Bioeng. Biotechnol..

[58] Sanjeewa K.K.A., Jayawardena T.U., Kim S.-Y., Kim H.-S., Ahn G., Kim J., Jeon Y.-J. (2019). Fucoidan Isolated from Invasive Sargassum Horneri Inhibit LPS-Induced Inflammation via Blocking NF-ΚB and MAPK Pathways. Algal Res..

[59] Hu X., Li J., Fu M., Zhao X., Wang W. (2021). The JAK/STAT Signaling Pathway: From Bench to Clinic. Signal Transduct. Target Ther..

[60] Kiu H., Nicholson S.E. (2012). Biology and Significance of the JAK/STAT Signalling Pathways. Growth Factors.

[61] Wu M., Song D., Li H., Yang Y., Ma X., Deng S., Ren C., Shu X. (2019). Negative Regulators of STAT3 Signaling Pathway in Cancers. Cancer Manag. Res..

[62] Seif F., Khoshmirsafa M., Aazami H., Mohsenzadegan M., Sedighi G., Bahar M. (2017). The Role of JAK-STAT Signaling Pathway and Its Regulators in the Fate of T Helper Cells. Cell Commun. Signal..

[63] Hemmings B.A., Restuccia D.F. (2012). PI3K-PKB/Akt Pathway. Cold Spring Harb. Perspect. Biol..

[64] He X., Li Y., Deng B., Lin A., Zhang G., Ma M., Wang Y., Yang Y., Kang X. (2022). The PI3K/AKT signalling pathway in inflammation, cell death and glial scar formation after traumatic spinal cord injury: Mechanisms and therapeutic opportunities. Cell Prolif..

[65] Lee H.-J., Dang H.-T., Kang G.-J., Yang E.-J., Park S.-S., Yoon W.-J., Jung J.H., Kang H.-K., Yoo E.-S. (2009). Two Enone Fatty Acids Isolated from *Gracilaria verrucosa* Suppress the Production of Inflammatory Mediators by Down-Regulating NF-ΚB and STAT1 Activity in Lipopolysaccharide-Stimulated RAW 264.7 Cells. Arch. Pharm. Res..

[66] Kobayashi E.H., Suzuki T., Funayama R., Nagashima T., Hayashi M., Sekine H., Tanaka N., Moriguchi T., Motohashi H., Nakayama K. (2016). Nrf2 Suppresses Macrophage Inflammatory Response by Blocking Proinflammatory Cytokine Transcription. Nat. Commun..

[67] Tebay L.E., Robertson H., Durant S.T., Vitale S.R., Penning T.M., Dinkova-Kostova A.T., Hayes J.D. (2015). Mechanisms of Activation of the Transcription Factor Nrf2 by Redox Stressors, Nutrient Cues, and Energy Status and the Pathways through Which It Attenuates Degenerative Disease. Free Radic. Biol. Med..

[68] Alcaraz M.J., Fernández P., Guillén M.I. (2003). Anti-Inflammatory Actions of the Heme Oxygenase-1 Pathway. Curr. Pharm. Des..

[69] Laskin D.L., Sunil V.R., Gardner C.R., Laskin J.D. (2011). Macrophages and Tissue Injury: Agents of Defense or Destruction?. Annu. Rev. Pharmacol. Toxicol..

[70] Saha S., Buttari B., Panieri E., Profumo E., Saso L. (2020). An Overview of Nrf2 Signaling Pathway and Its Role in Inflammation. Molecules.

[71] Kang H., Lee Y., Bae M., Park Y.-K., Lee J.-Y. (2020). Astaxanthin Inhibits Alcohol-Induced Inflammation and Oxidative Stress in Macrophages in a Sirtuin 1-Dependent Manner. J. Nutr. Biochem..

[72] Sirajunnisa A.R., Surendhiran D., Kozani P.S., Kozani P.S., Hamidi M., Cabrera-Barjas G., Delattre C. (2021). An Overview on the Role of Microalgal Metabolites and Pigments in Apoptosis Induction against Copious Diseases. Algal Res..

[73] Canton M., Sánchez-Rodríguez R., Spera I., Venegas F.C., Favia M., Viola A., Castegna A. (2021). Reactive Oxygen Species in Macrophages: Sources and Targets. Front. Immunol..

[74] Kim M.-B., Kang H., Li Y., Park Y.-K., Lee J.-Y. (2021). Fucoxanthin Inhibits Lipopolysaccharide-Induced Inflammation and Oxidative Stress by Activating Nuclear Factor E2-Related Factor 2 via the Phosphatidylinositol 3-Kinase/AKT Pathway in Macrophages. Eur. J. Nutr..

[75] Din N.A.S., Mohd Alayudin A.S., Sofian-Seng N.-S., Rahman H.A., Razali N.S.M., Lim S.J., Mustapha W.A.W. (2022). Brown Algae as Functional Food Source of Fucoxanthin: A Review. Foods.

[76] Wang X., Ma J., Bai X., Yan H., Qin C., Ren D. (2019). Antioxidant Properties of Astaxanthin Produced by Cofermentation between Spirulina Platensis and Recombinant *Saccharomyces cerevisiae* against Mouse Macrophage RAW 264.7 Damaged by H_2_O_2_. Food Bioprod. Process..

[77] Elmore S. (2007). Apoptosis: A Review of Programmed Cell Death. Toxicol. Pathol..

[78] DeNardo D.G., Ruffell B. (2019). Macrophages as Regulators of Tumour Immunity and Immunotherapy. Nat. Rev. Immunol..

[79] Mantovani A., Marchesi F., Malesci A., Laghi L., Allavena P. (2017). Tumour-Associated Macrophages as Treatment Targets in Oncology. Nat. Rev. Clin. Oncol..

[80] Seimon T., Tabas I. (2009). Mechanisms and Consequences of Macrophage Apoptosis in Atherosclerosis. J. Lipid Res..

[81] Hanahan D., Weinberg R.A. (2011). Hallmarks of Cancer: The Next Generation. Cell.

[82] Fazeela Mahaboob Begum S.M., Hemalatha S. (2020). Phytoconstituents from *Gelidiella acerosa* Induce Apoptosis by Regulating Bax, Bcl2 Expression in A549 Cells. Biocatal. Agric. Biotechnol..

[83] Han M.H., Lee D., Jeong J., Hong S., Choi I., Cha H., Kim S., Kim H., Park C., Kim G. (2017). Fucoidan Induces ROS-Dependent Apoptosis in 5637 Human Bladder Cancer Cells by Downregulating Telomerase Activity via Inactivation of the PI3K/Akt Signaling Pathway. Drug Dev. Res..

[84] Lee H.-E., Choi E.-S., Shin J.-A., Lee S.-O., Park K.-S., Cho N.-P., Cho S.-D. (2014). Fucoidan Induces Caspase-Dependent Apoptosis in MC3 Human Mucoepidermoid Carcinoma Cells. Exp. Ther. Med..

[85] Pradhan B., Patra S., Nayak R., Behera C., Dash S.R., Nayak S., Sahu B.B., Bhutia S.K., Jena M. (2020). Multifunctional Role of Fucoidan, Sulfated Polysaccharides in Human Health and Disease: A Journey under the Sea in Pursuit of Potent Therapeutic Agents. Int. J. Biol. Macromol..

[86] Aisa Y., Miyakawa Y., Nakazato T., Shibata H., Saito K., Ikeda Y., Kizaki M. (2005). Fucoidan Induces Apoptosis of Human HS-Sultan Cells Accompanied by Activation of Caspase-3 and down-Regulation of ERK Pathways. Am. J. Hematol..

[87] Teruya T., Konishi T., Uechi S., Tamaki H., Tako M. (2007). Anti-Proliferative Activity of Oversulfated Fucoidan from Commercially Cultured *Cladosiphon okamuranus* TOKIDA in U937 Cells. Int. J. Biol. Macromol..

[88] Nakamura T., Suzuki H., Wada Y., Kodama T., Doi T. (2006). Fucoidan Induces Nitric Oxide Production via P38 Mitogen-Activated Protein Kinase and NF-ΚB-Dependent Signaling Pathways through Macrophage Scavenger Receptors. Biochem. Biophys. Res. Commun..

[89] Hang D., Choi H.-S., Kang S.-C., Kim K.-R., Sohn E.-S., Kim M.-H., Pyo S., Son E.-W. (2005). Effects of Fucoidan on NO Production and Phagocytosis of Macrophages and the Proliferation of Neuron Cells. Prev. Nutr. Food Sci..

[90] Suganya A.M., Sanjivkumar M., Chandran M.N., Palavesam A., Immanuel G. (2016). Pharmacological Importance of Sulphated Polysaccharide Carrageenan from Red Seaweed *Kappaphycus alvarezii* in Comparison with Commercial Carrageenan. Biomed. Pharmacother..

[91] Ghannam A., Murad H., Jazzara M., Odeh A., Allaf A.W. (2018). Isolation, Structural Characterization, and Antiproliferative Activity of Phycocolloids from the Red Seaweed Laurencia Papillosa on MCF-7 Human Breast Cancer Cells. Int. J. Biol. Macromol..

[92] Khotimchenko M., Tiasto V., Kalitnik A., Begun M., Khotimchenko R., Leonteva E., Bryukhovetskiy I., Khotimchenko Y. (2020). Antitumor Potential of Carrageenans from Marine Red Algae. Carbohydr. Polym..

[93] Anand J., Sathuvan M., Babu G.V., Sakthivel M., Palani P., Nagaraj S. (2018). Bioactive Potential and Composition Analysis of Sulfated Polysaccharide from *Acanthophora spicifera* (Vahl) Borgeson. Int. J. Biol. Macromol..

[94] Ramaswamy U., Velusamy S., Devaraj N.S. (2020). Cytotoxicity and Apoptosis of Human Colon Carcinoma Cell Line (HT29 Cells), Treated with Methanolic Extract of Chlorococcum Humicola. Biotechnological Applications in Human Health.

[95] Ryu M.J., Kim A.D., Kang K.A., Chung H.S., Kim H.S., Suh I.S., Chang W.Y., Hyun J.W. (2013). The Green Algae *Ulva fasciata* Delile Extract Induces Apoptotic Cell Death in Human Colon Cancer Cells. In Vitro Cell Dev. Biol. Anim..

[96] Al Monla R., Dassouki Z., Kouzayha A., Salma Y., Gali-Muhtasib H., Mawlawi H. (2020). The Cytotoxic and Apoptotic Effects of the Brown Algae *Colpomenia sinuosa* Are Mediated by the Generation of Reactive Oxygen Species. Molecules.

[97] Leiro J.M., Castro R., Arranz J.A., Lamas J. (2007). Immunomodulating Activities of Acidic Sulphated Polysaccharides Obtained from the Seaweed *Ulva rigida* C. Agardh. Int. Immunopharmacol..

[98] Barbalace M.C., Malaguti M., Giusti L., Lucacchini A., Hrelia S., Angeloni C. (2019). Anti-Inflammatory Activities of Marine Algae in Neurodegenerative Diseases. Int. J. Mol. Sci..

[99] Olasehinde T., Olaniran A., Okoh A. (2017). Therapeutic Potentials of Microalgae in the Treatment of Alzheimer’s Disease. Molecules.

[100] Miguel S.P., Ribeiro M.P., Otero A., Coutinho P. (2021). Application of Microalgae and Microalgal Bioactive Compounds in Skin Regeneration. Algal Res..

[101] Ognistaia A.V., Markina Z.V., Orlova T.Y. (2022). Antimicrobial Activity of Marine Microalgae. Russ. J. Mar. Biol..

[102] Wong J.F., Hong H.J., Foo S.C., Yap M.K.K., Tan J.W. (2022). A Review on Current and Future Advancements for Commercialized Microalgae Species. Food Sci. Hum. Wellness.

[103] Shannon E., Abu-Ghannam N. (2016). Antibacterial Derivatives of Marine Algae: An Overview of Pharmacological Mechanisms and Applications. Mar. Drugs.

[104] Chu C.Y., Liao W.R., Huang R., Lin L.P. (2004). Haemagglutinating and Antibiotic Activities of Freshwater Microalgae. World J. Microbiol. Biotechnol..

[105] Frazzini S., Scaglia E., Dell’Anno M., Reggi S., Panseri S., Giromini C., Lanzoni D., Sgoifo Rossi C.A., Rossi L. (2022). Antioxidant and Antimicrobial Activity of Algal and Cyanobacterial Extracts: An In Vitro Study. Antioxidants.

[106] Dell’Anno M., Sotira S., Rebucci R., Reggi S., Castiglioni B., Rossi L. (2020). *In Vitro* Evaluation of Antimicrobial and Antioxidant Activities of Algal Extracts. Ital. J. Anim. Sci..

[107] Wong B.T., Park S., Kovanda L., He Y., Kim K., Xu S., Lingga C., Hejna M., Wall E., Sripathy R. (2022). Dietary Supplementation of Botanical Blends Enhanced Performance and Disease Resistance of Weaned Pigs Experimentally Infected with Enterotoxigenic *Escherichia Coli* F18. J. Anim. Sci..

[108] Rodrigues D., Alves C., Horta A., Pinteus S., Silva J., Culioli G., Thomas O., Pedrosa R. (2015). Antitumor and Antimicrobial Potential of Bromoditerpenes Isolated from the Red Alga, *Sphaerococcus coronopifolius*. Mar. Drugs.

[109] Beaulieu L., Bondu S., Doiron K., Rioux L.-E., Turgeon S.L. (2015). Characterization of Antibacterial Activity from Protein Hydrolysates of the Macroalga *Saccharina longicruris* and Identification of Peptides Implied in Bioactivity. J. Funct. Foods.

[110] Corino C., Modina S.C., Di Giancamillo A., Chiapparini S., Rossi R. (2019). Seaweeds in Pig Nutrition. Animals.

[111] Berri M., Slugocki C., Olivier M., Helloin E., Jacques I., Salmon H., Demais H., Le Goff M., Collen P.N. (2016). Marine-Sulfated Polysaccharides Extract of *Ulva armoricana* Green Algae Exhibits an Antimicrobial Activity and Stimulates Cytokine Expression by Intestinal Epithelial Cells. J. Appl. Phycol..

[112] Mcelwain T.F., Thumbi S.M. (2017). Animal Pathogens and Their Impact on Animal Health, the Economy, Food Security, Food Safety and Public Health. Rev. Sci. Tech..

[113] Niemi J.K. (2021). The Economic Cost of Bacterial Infections. Advancements and Technologies in Pig and Poultry Bacterial Disease Control.

[114] Hejna M., Dell’Anno M., Liu Y., Rossi L., Aksmann A., Pogorzelski G., Jóźwik A. (2024). Assessment of the Antibacterial and Antioxidant Activities of Seaweed-Derived Extracts. Sci. Rep..

[115] Bhowmick S., Mazumdar A., Moulick A., Adam V. (2020). Algal Metabolites: An Inevitable Substitute for Antibiotics. Biotechnol. Adv..

[116] Brown E.D., Wright G.D. (2016). Antibacterial Drug Discovery in the Resistance Era. Nature.

[117] Santoyo S., Rodríguez-Meizoso I., Cifuentes A., Jaime L., García-Blairsy Reina G., Señorans F.J., Ibáñez E. (2009). Green Processes Based on the Extraction with Pressurized Fluids to Obtain Potent Antimicrobials from *Haematococcus pluvialis* Microalgae. LWT Food Sci. Technol..

[118] Syed S., Arasu A., Ponnuswamy I. (2015). The Uses of *Chlorella vulgaris* as Antimicrobial Agent and as a Diet: The Presence of Bio-Active Compounds Which Caters the Vitamins, Minerals in General. Int. J. Bio-Sci. Bio-Technol..

[119] Molina-Cárdenas C.A., Sánchez-Saavedra M.d.P., Lizárraga-Partida M.L. (2014). Inhibition of Pathogenic Vibrio by the Microalgae *Isochrysis galbana*. J. Appl. Phycol..

[120] Herrero M., Ibáñez E., Cifuentes A., Reglero G., Santoyo S. (2006). *Dunaliella salina* Microalga Pressurized Liquid Extracts as Potential Antimicrobials. J. Food Prot..

[121] Catarina Guedes A., Barbosa C.R., Amaro H.M., Pereira C.I., Xavier Malcata F. (2011). Microalgal and Cyanobacterial Cell Extracts for Use as Natural Antibacterial Additives against Food Pathogens. Int. J. Food Sci. Technol..

[122] Desbois A.P., Mearns-Spragg A., Smith V.J. (2009). A Fatty Acid from the Diatom *Phaeodactylum tricornutum* Is Antibacterial against Diverse Bacteria Including Multi-Resistant *Staphylococcus aureus* (MRSA). Mar. Biotechnol..

[123] Bai V.D.M., Krishnakumar S. (2013). Evaluation of Antimicrobial Metabolites from Marine Microalgae *Tetraselmis suecica* Using Gas Chromatography–Mass Spectrometry (GC–MS) Analysis. Int. J. Pharm. Pharm. Sci..

[124] Menaa F., Wijesinghe U., Thiripuranathar G., Althobaiti N.A., Albalawi A.E., Khan B.A., Menaa B. (2021). Marine Algae-Derived Bioactive Compounds: A New Wave of Nanodrugs?. Mar. Drugs.

[125] Abu-Ghannam N., Rajauria G. (2013). Antimicrobial Activity of Compounds Isolated from Algae. Functional Ingredients from Algae for Foods and Nutraceuticals.

[126] Karpiński T.M., Adamczak A. (2019). Fucoxanthin—An Antibacterial Carotenoid. Antioxidants.

[127] Lane A.L., Stout E.P., Lin A.-S., Prudhomme J., Le Roch K., Fairchild C.R., Franzblau S.G., Hay M.E., Aalbersberg W., Kubanek J. (2009). Antimalarial Bromophycolides J–Q from the Fijian Red Alga *Callophycus serratus*. J. Org. Chem..

[128] Bassols A., Costa C., Eckersall P.D., Osada J., Sabrià J., Tibau J. (2014). The Pig as an Animal Model for Human Pathologies: A Proteomics Perspective. Proteom. Clin. Appl..

[129] Meurens F., Summerfield A., Nauwynck H., Saif L., Gerdts V. (2012). The Pig: A Model for Human Infectious Diseases. Trends Microbiol..

[130] Walters E.M., Wells K.D., Bryda E.C., Schommer S., Prather R.S. (2017). Swine Models, Genomic Tools and Services to Enhance Our Understanding of Human Health and Diseases. Lab. Anim..

[131] Walters E.M., Prather R.S. (2013). Advancing Swine Models for Human Health and Diseases. Mo. Med..

[132] Rose E.C., Blikslager A.T., Ziegler A.L. (2022). Porcine Models of the Intestinal Microbiota: The Translational Key to Understanding How Gut Commensals Contribute to Gastrointestinal Disease. Front. Vet. Sci..

[133] Singh V.K., Thrall K.D., Hauer-Jensen M. (2016). Minipigs as Models in Drug Discovery. Expert Opin. Drug Discov..

[134] Kleinert M., Clemmensen C., Hofmann S.M., Moore M.C., Renner S., Woods S.C., Huypens P., Beckers J., de Angelis M.H., Schürmann A. (2018). Animal Models of Obesity and Diabetes Mellitus. Nat. Rev. Endocrinol..

[135] Verma N., Rettenmeier A.W., Schmitz-Spanke S. (2011). Recent Advances in the Use of *Sus scrofa* (Pig) as a Model System for Proteomic Studies. Proteomics.

[136] Osada H., Murata K., Masumoto H. (2023). Large Animal Models in Cardiovascular Research. Animal Models and Experimental Research in Medicine.

[137] Babich O., Sukhikh S., Larina V., Kalashnikova O., Kashirskikh E., Prosekov A., Noskova S., Ivanova S., Fendri I., Smaoui S. (2022). Algae: Study of Edible and Biologically Active Fractions, Their Properties and Applications. Plants.

[138] Shalaby E.A. (2011). Algae as Promising Organisms for Environment and Health. Plant Signal. Behav..

[139] Kim K., Ehrlich A., Perng V., Chase J.A., Raybould H., Li X., Atwill E.R., Whelan R., Sokale A., Liu Y. (2019). Algae-Derived β-Glucan Enhanced Gut Health and Immune Responses of Weaned Pigs Experimentally Infected with a Pathogenic *E. coli*. Anim. Feed. Sci. Technol..

[140] Manor M.L., Kim J., Derksen T.J., Schwartz R.L., Roneker C.A., Bhatnagar R.S., Lei X.G. (2017). Defatted Microalgae Serve as a Dual Dietary Source of Highly Bioavailable Iron and Protein in an Anemic Pig Model. Algal Res..

[141] Smith A.G., O’Doherty J.V., Reilly P., Ryan M.T., Bahar B., Sweeney T. (2011). The Effects of Laminarin Derived from *Laminaria digitata* on Measurements of Gut Health: Selected Bacterial Populations, Intestinal Fermentation, Mucin Gene Expression and Cytokine Gene Expression in the Pig. Br. J. Nutr..

[142] Zheng L.-X., Chen X.-Q., Cheong K.-L. (2020). Current Trends in Marine Algae Polysaccharides: The Digestive Tract, Microbial Catabolism, and Prebiotic Potential. Int. J. Biol. Macromol..

[143] Shannon E., Conlon M., Hayes M. (2021). Seaweed Components as Potential Modulators of the Gut Microbiota. Mar. Drugs.

[144] Zhang H., Jiang F., Zhang J., Wang W., Li L., Yan J. (2022). Modulatory Effects of Polysaccharides from Plants, Marine Algae and Edible Mushrooms on Gut Microbiota and Related Health Benefits: A Review. Int. J. Biol. Macromol..

[145] Cheong K.-L., Yu B., Chen J., Zhong S. (2022). A Comprehensive Review of the Cardioprotective Effect of Marine Algae Polysaccharide on the Gut Microbiota. Foods.

[146] Liyanage N.M., Nagahawatta D.P., Jayawardena T.U., Jeon Y.-J. (2023). The Role of Seaweed Polysaccharides in Gastrointestinal Health: Protective Effect against Inflammatory Bowel Disease. Life.

[147] Singh R.P., Bhaiyya R., Khandare K., Tingirikari J.M.R. (2022). Macroalgal Dietary Glycans: Potential Source for Human Gut Bacteria and Enhancing Immune System for Better Health. Crit. Rev. Food Sci. Nutr..

